# Rhombohedral 3R MoS_2_ Polytype: A Promising Fundamental Material for Next‐Generation Device Applications

**DOI:** 10.1002/smll.202504644

**Published:** 2025-06-16

**Authors:** Prabhukrupa Chinmay Kumar, Sang Mun Jeong, Ramakanta Naik, Chandra Sekhar Rout

**Affiliations:** ^1^ Department of Engineering and Materials Physics Institute of Chemical Technology‐Indian Oil Odisha Campus Bhubaneswar 751013 India; ^2^ Department of Chemical Engineering Chungbuk National University Cheongju Chungbuk 28644 Republic of Korea; ^3^ Advanced Energy Research Institute Chungbuk National University Cheongju Chungbuk 28644 Republic of Korea; ^4^ Centre for Nano and Material Sciences, Jain (Deemed–to–be University) Jain Global Campus Kanakapura Road Bangalore Karnataka 562112 India

**Keywords:** 2D materials, 3R phase, layered structure, MoS_2_, polymorphs

## Abstract

The 3R polymorph of MoS_2_ demonstrates remarkable versatility across multiple fields, from energy storage and environmental remediation to optoelectronics and quantum technologies. Its unique properties, such as broken inversion symmetry, high surface area, and robust valley coherence, make it a promising material for addressing current technological challenges. As research continues to uncover its potential, 3R‐MoS_2_ is poise to play a pivotal role in advancing next‐generation devices and applications. This review highlights the growing interest in the 3R polytype of molybdenum disulfide (MoS_2_), a metastable yet thermodynamically stable phase with unique ABC stacking and broken inversion symmetry. In comparison to the widely studied 2H phase, 3R‐MoS_2_ exhibits distinctive structural, electronic, and optical properties, making it highly suitable for various next‐generation device applications in the field of quantum technologies, energy storage, catalysis, and optoelectronics. The review covers synthesis methods, structural and electronic characteristics, and various application domains 3R‐MoS_2_ while addressing challenges such as limited exploration, synthesis difficulties, and underutilized properties. Future prospects and directions for research are also outlined to encourage broader investigation and technological implementation.

## Graphical abstract

Different synthesis methods properties and applications the 3R‐MoS_2_ are briefly described with necessary figures, plots, and tables as well as the future scopes in the field of research on these materials, are elaborately discussed in this review.

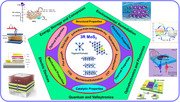

## 1 Introduction

Molybdenum disulfide MoS_2_ as a well‐known transition metal dichalcogenide (TMD), are layered materials with substantial in‐plane ionic‐covalent bonds and feeble out‐of‐plane van der Waals interactions.^[^ ^1^, ^2^, ^3^, ^4^ ^]^ These materials can exist in different polymorphic structures, including the commonly known 1T, 2H phase, and the less common 3R phase.^[^ ^5^, ^6^ ^]^ The rhombohedral 3R polytype of MoS_2_ has garnered momentous consideration in recent years due to its unique structural and electronic properties and potential applications, which distinguish it from other polytypes such as the hexagonal (2H) and tetragonal (1T) forms.^[^ ^7^, ^8^ ^]^ This polytype exhibits distinct characteristics that differentiate it from the more common 2H phase, including enhanced ferroelectricity, superior electrocatalytic performance, and robust valley polarization. The 3R MoS_2_ exhibits an ABC stacking arrangement, which is characterized by a lack of inversion symmetry, a feature that is crucial for various applications in photonics and optoelectronics.^[^ ^9^ ^]^ This polytype is thermodynamically stable and presents a bandgap that can be tuned based on the number of layers, ranging from approximately 1.2 eV in bulk to around 1.8 eV in monolayer forms.^[^ ^8^ ^]^ The exclusive stacking and electronic properties of 3R MoS_2_ make it an attractive candidate for a variety of device applications, including transistors, photodetectors, and electrocatalysts.

The structural characteristics of 3R MoS_2_, particularly its rhombohedral symmetry, contribute to its discrete electronic properties. Unlike the 2H phase, which is centrosymmetric, the 3R phase allows for valley‐dependent spin polarization, making it a promising material for spintronic applications.^[^ ^10^ ^]^ This property is particularly relevant in the context of developing devices that leverage spin‐polarized currents, which can lead to enhanced performance in information processing and storage technologies. Furthermore, the layered nature of MoS_2_, combined with its weak van der Waals interactions between layers, facilitates the fabrication of heterostructures that can be engineered for specific functionalities.^[^ ^11^, ^12^ ^]^ In the realm of electrochemical applications, 3R MoS_2_ has shown promise as an electrocatalyst for hydrogen evolution reactions (HER). Its unique electronic structure provides a high density of active sites, which can enhance the efficiency of electrochemical reactions.^[^ ^13^ ^]^ Studies have demonstrated that 3R‐MoS_2_ can outperform other polytypes in terms of catalytic activity, making it a suitable candidate for energy conversion technologies.^[^ ^14^ ^]^ Additionally, the ability to intercalate ions within its layered structure allows for its use in energy storage devices, such as supercapacitors and batteries, where rapid ion transport is essential.^[^ ^15^ ^]^

The synthesis of 3R‐MoS_2_ can be achieved through various methods, including hydrothermal and solvothermal techniques, which allow for control over the morphology and structural properties of the resulting material.^[^ ^16^ ^]^ The ability to tailor the synthesis parameters enables the production of 3R‐MoS_2_ with specific characteristics that can be optimized for specific applications. 3R‐MoS_2_ synthesis often requires crystal growth from screw dislocations.^[^ ^17^, ^18^, ^19^ ^]^ Chemical vapor deposition (CVD), chemical vapor transport (CVT), flux growth, and exfoliation techniques are the most commonly used methods for preparing 3R‐MoS_2_ materials.^[^ ^20^ ^]^ In their review, Strachan et al. elaborately covered a chemically focused historical and structural overview of 3R‐MoS_2_, emphasizing synthesis pathways, growth mechanisms, polytype evolution, and mischaracterization issues. The study primarily concentrates on crystallographic definitions, flux‐based growth, natural occurrence, and polytypic structures, with a strong emphasis on fundamental crystallography and synthesis history.^[^ ^9^ ^]^

In terms of device applicability, 3R‐MoS_2_ has been integrated into various electronic and optoelectronic devices, including dual‐gate transistors that leverage its high mobility and tunable bandgap.^[^ ^21^ ^]^ The incorporation of 3R‐MoS_2_ into van der Waals heterostructures has also been explored, with promising results in photodetection across a wide spectral range, from visible to near‐infrared light.^[^ ^22^ ^]^ The ability to efficiently catalyze both the hydrogen evolution and oxygen evolution reactions (OER) positions 3R‐MoS_2_ as a key material in the development of sustainable energy solutions. Its stability and performance metrics, such as overpotential and exchange current density, have been extensively studied, revealing its viability as an alternative to traditional noble metal catalysts. Various types of biosensors utilizing MoS_2‐_based materials have been developed, offering valuable insights and motivation for improving sensing performance.^[^ ^23^ ^]^ The 3R MoS_2_ polytype demonstrates room‐temperature switchable ferroelectricity, which is crucial for developing ultrathin computing‐in‐memory devices.^[^ ^24^ ^]^ The 3R polytype supports robust valley polarization, projecting it as a capable candidate for valleytronic applications.^[^ ^25^ ^]^ In contrast, while the 3R polytype shows significant advantages, the 2H phase remains more widely studied and utilized in existing applications due to its established synthesis methods, potentially limiting the immediate adoption of 3R MoS_2_ in commercial technologies, indicating a need for further exploration of 3R MoS_2_’s potential in practical applications.

**Figure 1:**
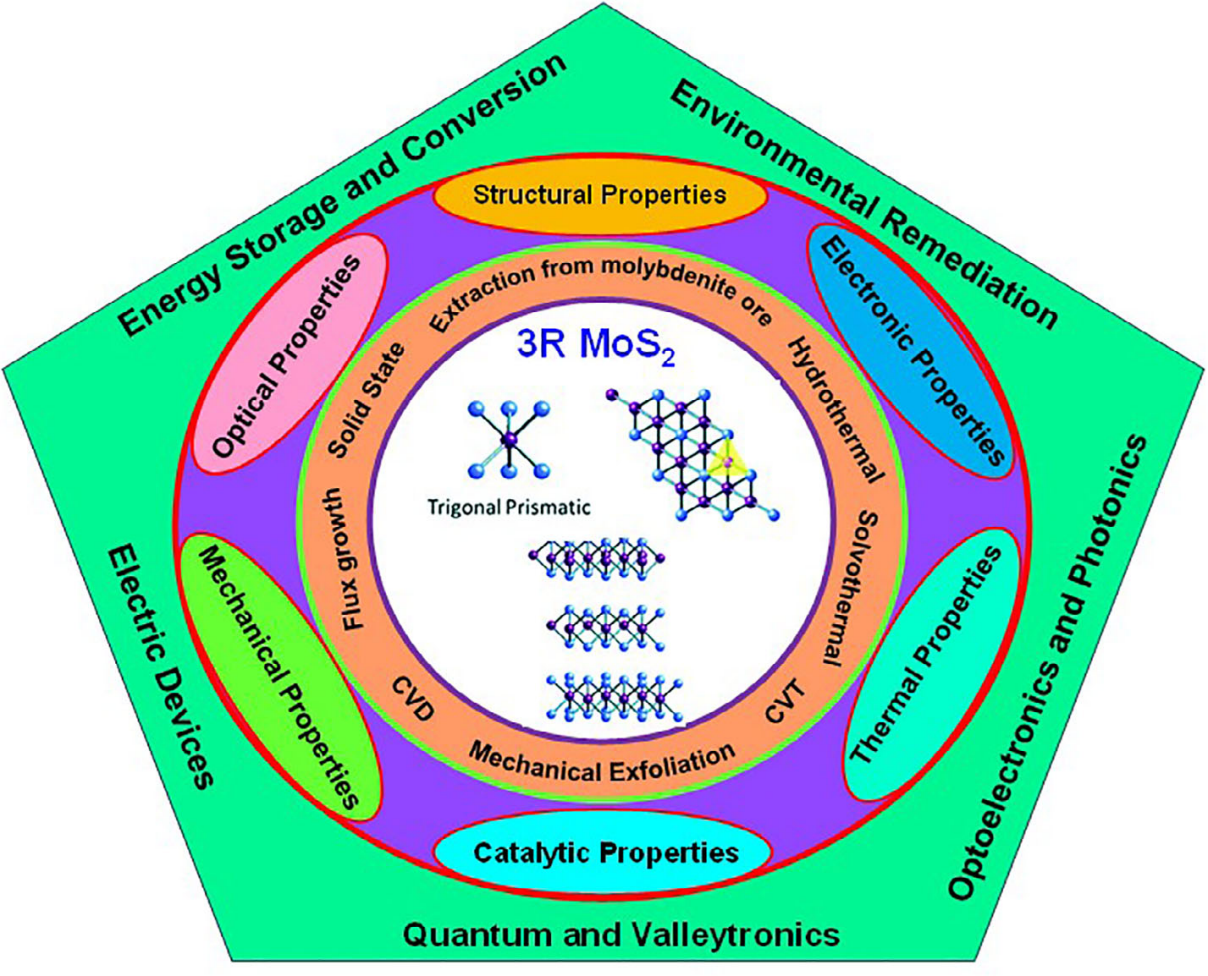
Schematic representation of the different synthesis procedures, properties, and applications of 3R‐MoS_2_.

Here in this review, we have categorized the synthesis, structure, property, and application framework, offering schematic illustrations and comparative tables to guide researchers toward practical and scalable implementation of 3R‐MoS_2_. Through classifications, we have systematically reviewed top‐down and bottom‐up synthesis methods, highlighting their influence on morphology and functional performance with discussions regarding the advanced characterizations, relevant to next‐generation devices. It explores emerging functionalities, such as valley coherence, second harmonic generation, ferroelectric switching, photodetection, and piezoelectric effects, that are not covered in Strachan et al.’s work. Our aim in this review work is to emphasize device‐oriented applications, including quantum technologies, energy storage, catalysis, optoelectronics, environmental remediation, and piezoelectric nanodevices. Our work extends beyond the chemical and structural background to offer a multidisciplinary view tailored to modern functional devices, making it a valuable and complementary contribution to the existing literature. The complete review content is schematically represented in **Figure** **1** of the different preparation methods, properties, and applications of 3R‐MoS_2_.

## 2 Typical Structure and Electronic Features

### 2.1 Structural Features

The structural features of the 3R‐MoS_2_ phase include the following elements:

#### 2.1.1 Crystal Structure

**Figure 2:**
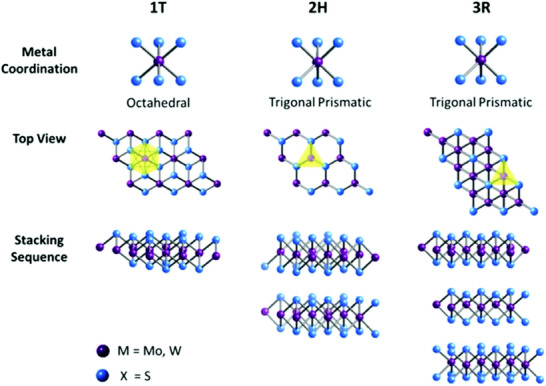
Metal co‐ordinations and stacking sequences of tetragonal symmetry (1T), hexagonal symmetry (2H), and rhombohedral symmetry (3R) of MoS_2_ material. Reproduced with permission from the Royal Society of Chemistry.^[^ ^35^ ^]^

3R‐MoS_2_ is characterized by a rhombohedral crystal structure. It lacks an inversion center, which distinguishes it from the more common 2H‐MoS_2_ phase, which has a hexagonal structure. Its structure has an R3m space group with stacking order AbABcBCaC *a* = 3.17 Å and *c* = 18.38 Å.^[^ ^19^ ^]^ 3R‐MoS_2_ is composed of layered sheets made of covalently bonded Mo–S trigonal prisms, known as “sandwich layers,”^[^ ^26^, ^27^ ^]^ which are more compact than various TMDs because of the Mo atom's smaller size.^[^ ^28^, ^29^ ^]^ The electronic structure, metal coordination, and stacking order of different polymorphs of MoS_2_ are depicted in **Figure** **2**.

#### 2.1.2 Layer Arrangement

The 3R structure is defined by a specific stacking arrangement of the molybdenum (Mo) and sulfur (S) layers. This sequence can influence the electronic and optical properties of the material. In 3R‐MoS_2_, these layers are stacked in a manner that creates a unique set of properties compared to its polytypes, particularly in how they interact with external stimuli.^[^ ^30^ ^]^

#### 2.1.3 High Density of Edge Sites

The morphology of 3R‐MoS_2_ often leads to a high density of edge sites, which are crucial for catalytic activity, particularly in reactions such as the HER.^[^ ^31^ ^]^ These edge sites are typically more reactive than basal plane sites and contribute significantly to the material's catalytic performance.

#### 2.1.4 Unique Morphologies

3R‐MoS_2_ can exhibit distinct morphologies, such as triangular pyramids grown from screw dislocations.^[^ ^32^ ^]^ These morphologies result in surfaces that are rich in edge sites, enhancing their potential use in various chemical reactions and applications.^[^ ^33^ ^]^

#### 2.1.5 Mechanical Properties

The 3R phase is described as having properties such as malleability and a slightly higher density compared to the 2H phase (5.009 g cm^−3^ for 3R compared to 4.995 g cm^−3^ for 2H), affecting its processing and utilization.^[^ ^34^ ^]^

#### 2.1.6 Response to Strain

The piezoelectric properties of 3R‐MoS_2_ exhibit nonlinearity as a function of the number of layers,^[^ ^32^ ^]^ suggesting that the material can respond effectively to mechanical strain, which can be leveraged for energy conversion applications.

Overall, the unique structural features of 3R‐MoS_2_ not only contribute to its physical properties but also open avenues for various applications, from catalysis to sensor technology. Further studies into these structural characteristics could lead to optimized performance in practical uses.

### 2.2 Electronic Features

The electronic features of the 3R‐MoS_2_ phase include several important characteristics that differentiate it from other polytypes such as 2H‐MoS_2_:

#### 2.2.1 Tunable Band Gap

3R‐MoS_2_ exhibits a tunable band gap, which can be adjusted based on the number of layers and external conditions. For monolayer samples, the band gap is approximately 1.81 eV, while for bulk samples, it can be around 0.80 eV.^[^ ^36^ ^]^ This feature allows for the tailoring of electronic properties for specific applications in optoelectronics and photonic devices.

#### 2.2.2 Spin‐Orbit Coupling Effects

The presence of SOC in 3R MoS_2_ leads to significant splitting of electronic states, particularly near the valence band maximum (VBM) and conduction band minimum (CBM). This results in a clear separation of spin‐up and spin‐down states, which is advantageous for valleytronic applications where information can be stored using different valleys in the band structure.^[^ ^37^ ^]^

#### 2.2.3 High Piezoelectric Constant

The 3R‐MoS_2_ phase possesses a notably high piezoelectric constant (out‐of‐plane piezoelectric coefficient ranging from 0.7 to 1.5 pm V^−1^), which enables it to generate electrical charge in response to mechanical stress.^[^ ^22^ ^]^ This property makes it suitable for applications in sensors, actuators, flexible electronics, and energy harvesting technologies.

#### 2.2.4 Valley Polarization

Unlike the 2H phase, the 3R phase lacks inversion symmetry, enhancing its valley polarization properties. This characteristic allows for easier detection of valley polarization through circularly polarized photoluminescence (PL), making it a promising material for applications in valley/spin physics.^[^ ^25^, ^38^ ^]^

#### 2.2.5 Layer Coupling Sensitivity

The electronic properties of 3R MoS_2_ are sensitive to layer coupling and stacking order. Different stacking configurations (e.g., ABA vs. ABC) can lead to variations in electronic band structures, including distinct band‐splitting behaviors at different points in the Brillouin zone.^[^ ^39^ ^]^ This sensitivity allows for tailored electronic properties by adjusting the material's architecture.

#### 2.2.6 High Mobility and Low Contact Resistance

The 3R phase exhibits high electron mobility and low contact resistance, making it suitable for high‐performance electronic devices such as transistors.^[^ ^40^, ^41^ ^]^ Its layered structure allows for easy exfoliation into thin films, which is beneficial for creating nanoscale electronic components.

In summary, the unique electronic properties of 3R MoS_2_, such as its tunable band gap, significant SOC effects, enhanced valley polarization, sensitivity to layer stacking, high mobility, and piezoelectric characteristics, make it a versatile material with promising applications in advanced electronics, catalysis, and photonics. As research into its properties progresses, 3R‐MoS_2_ may reveal even more capabilities in fields such as energy conversion and storage.

## 3 Synthesis of 3R MoS_2_

**Figure 3:**
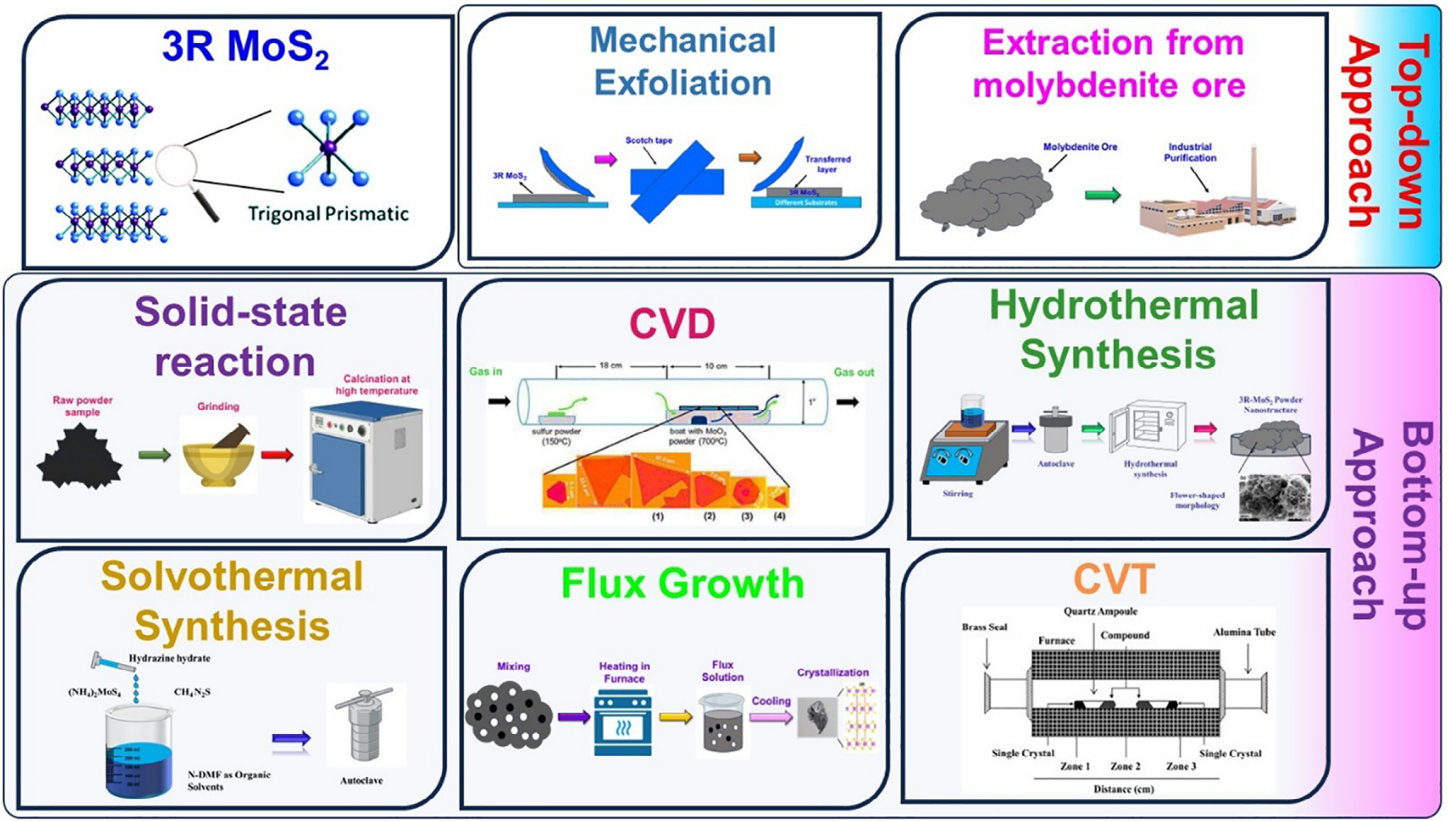
Schematic illustration of different synthesis methods of preparing 3R‐MoS_2_ nanomaterial.

In general, there are several methods to synthesize 3R‐MoS_2_ with different capabilities to regulate the phase, structure, and quality of the 3R‐MoS_2_, as well as advantages of scalability, purity, and structural regulation. The choice of synthesis method significantly impacts the material's properties and its suitability for various applications. As research continues, new techniques are being developed to improve the efficiency, scalability, and environmental impact of 3R‐MoS_2_ synthesis, paving the way for its broader adoption in advanced technological applications. The conventional 3R MoS_2_ synthesis methods were divided into two categories: top‐down and bottom‐up. The top‐down technique includes the mechanical exfoliation of bulk MoS_2_ into few‐layer or monolayer nanosheets. The common methods under this category are mechanical, liquid phase (LPE), electrochemical, and chemical exfoliation. A closely related bottom‐up approach takes molecular or atomic precursors to synthesize 3R MoS_2_. The most used techniques belonging to this category are CVD, hydrothermal/solvothermal synthesis, molecular beam epitaxy (MBE), thermal sulfurization, and template‐assisted growth. Each synthesis method is briefly discussed in the following section and represented through schematics in **Figure** **3**.

### 3.1 Top‐Down Approach

#### 3.1.1 Mechanical Exfoliation

This method, often referred to as the “Scotch tape method,” involves using adhesive tape to mechanically peel layers from a bulk crystal of MoS_2_. Although primarily effective for laboratory‐scale studies.^[^ ^42^ ^]^ It can produce monolayer and few‐layer 3R‐MoS_2_, which can then be transferred to different substrates (Si/SiO_2_).^[^ ^43^ ^]^ However, this approach does not allow for reliable control over phase composition or layer number. Additional mechanical techniques, including grinding, may also facilitate the development of 3R‐MoS_2_ domains within 2H‐MoS_2_ crystals, but similarly, these methods lack precision.^[^ ^44^, ^45^, ^46^ ^]^ Mechanical exfoliation is straightforward and can yield high‐quality monolayers or few‐layer materials. However, it is less scalable compared to other methods.

#### 3.1.2 Extraction from Molybdenite Ore

3R‐MoS_2_ can be synthesized through the extraction from naturally occurring molybdenite deposits. A substantial number of instances (exceeding 1000) have been documented globally that illustrate the extraction of 3R‐MoS_2_ samples, which surpasses the confines of this review to the present; nevertheless, pertinent meta‐analyses are provided to address this gap.^[^ ^47^, ^48^, ^49^ ^]^

There are a few other synthesis techniques that fall under the top‐down approach category, such as liquid phase exfoliation (LPE),^[^ ^50^, ^51^, ^52^, ^53^ ^]^ electrochemical deposition method, etc.,^[^ ^54^, ^55^ ^]^ but we decided not to include them here since the studies do not clearly explain how to synthesize 3R MoS_2_ using these techniques.

### 3.2 Bottom‐Up Approach

#### 3.2.1 Solid‐State Reaction

To synthesize 3R‐MoS_2_ flakes, a solid‐state reaction is performed in a horizontal furnace. Sodium molybdate is placed in a quartz boat and positioned at the tube's entrance, away from the furnace's hot zone. The furnace is heated to 850 °C under a nitrogen gas flow. Once stable, the sodium molybdate is moved into the hot zone, and nitrogen and hydrogen sulfide (H_2_S) gases are introduced at specific flow rates for 30 min to facilitate the reaction. Afterward, the boat is moved to a cooler zone, and the gas flow rates are adjusted for another 30 min to finalize the reaction. The resulting 3R‐MoS_2_ flakes are then separated from the reaction medium through sonication in water followed by filtration. This approach allows for the production of highly crystalline materials.^[^ ^56^ ^]^

#### 3.2.2 Chemical Vapor Deposition (CVD)

CVD is an effective technique for synthesizing high‐quality 3R‐MoS_2_. This process involves the precursors, i.e., “Mo” and “S,” in a gaseous state reacting to form 3R MoS_2_ deposition onto a substrate in a controlled environment, typically at high temperatures. CVD allows the production of large, relatively defect‐free sheets and facilitates control over layer number and morphology, which is essential for optimizing the properties of 3R‐MoS_2_. Through the manipulation of carrier gas flow dynamics, including the transition from continuous to pulsed mode, alongside the adjustment of growth conditions like temperature and gas flow, it is possible to obtain high‐quality 3R‐phase MoS_2_ with defined geometries in a controlled stacking configuration. This is important for ensuring the quality and uniformity of the crystals, which are essential for their application.

**Figure 4:**
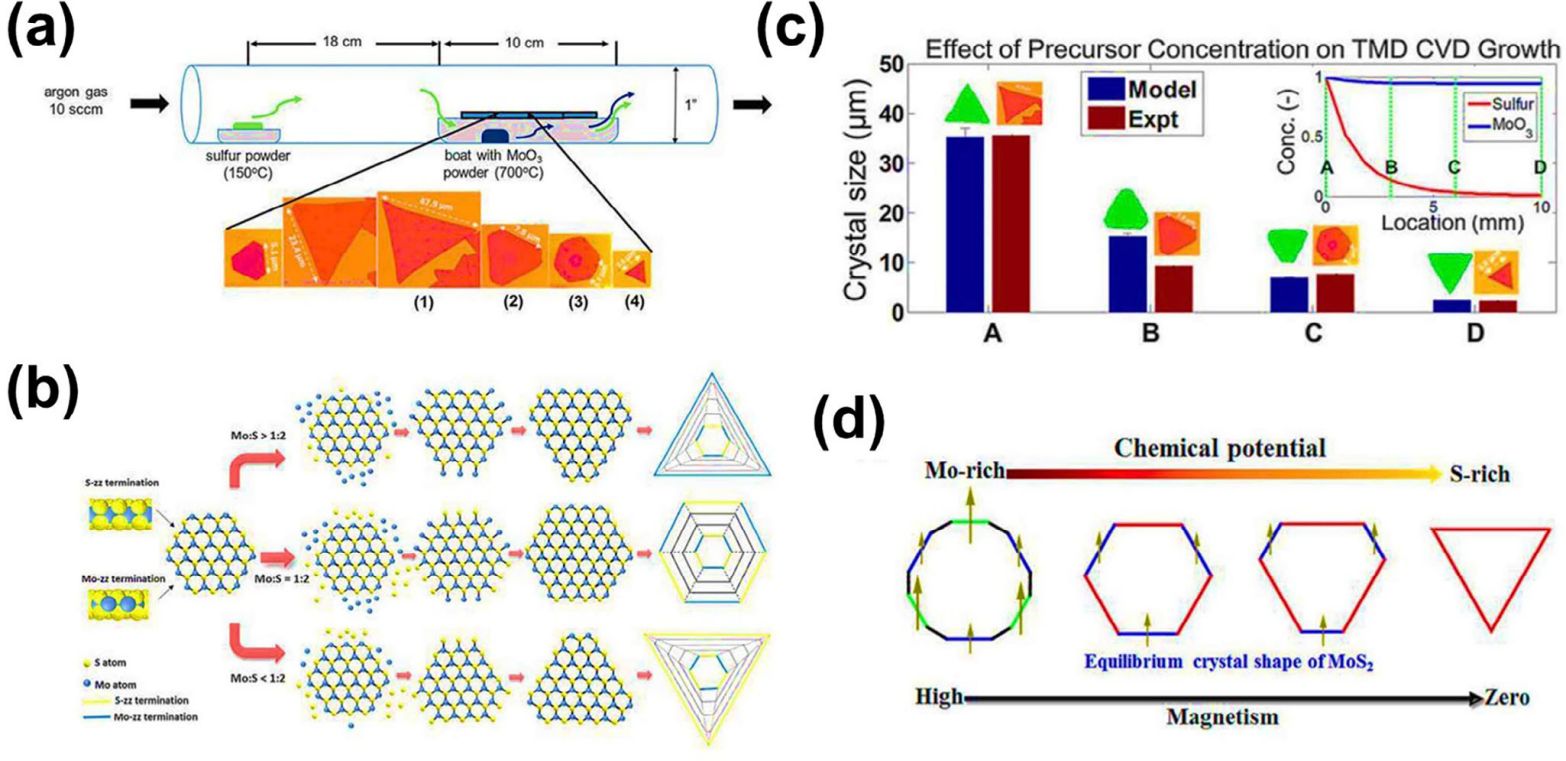
a) A schematic representation of MoS_2_ growth via chemical vapor deposition (CVD), accompanied by scanning electron microscopy (SEM) images showing the resulting structures, b) a statistical diagram illustrating how Mo:S atomic ratio influences the shape of MoS_2_ grains, c) predicted shapes and sizes of MoS_2_ crystals based on theoretical modeling, along with SEM images of growth patterns observed at various reactor locations, and d) analysis of magnetic properties and morphology of MoS_2_ domains under different chemical potential conditions.^[^ ^64^ ^]^

The experimental setup is shown in **Figure** **4**a, while Figure 4b illustrates how the Mo/S ratio influences the shape and edge structure of MoS_2_ crystals. This represents that higher Mo content forms triangular grains with Mo‐terminated (Mo‐zz) edges, and higher S content forms triangular grains with S‐terminated (S‐zz) edges.^[^ ^57^ ^]^ Figure 4c presents theoretical calculations explaining the impact of precursor concentration ratios, which were used to establish a systematic and reproducible growth method.^[^ ^58^ ^]^ Figure 4d highlights that precise control of the precursor ratio enables the tailoring of crystal shape and edge structure.^[^ ^59^ ^]^

In the CVD method for creating 3R‐MoS_2_, molybdenum trioxide (MoO_3_) and sulfur (S) powders are placed in a quartz tube within the CVD setup. The furnace is then heated to specific temperatures to drive the reaction. Initially, the temperature is elevated to 700 °C for the growth of the first layer, followed by an increase to 750 °C for the second layer. During growth, gases such as Ar and H_2_ are introduced at controlled flow rates to optimize the crystal growth environment, sometimes using a reverse flow of Ar/H_2_ during the second layer growth. The stacking order of the MoS_2_ layers is highly dependent on both the temperature and gas flow. Lower temperatures generally favor the 3R configuration, while higher temperatures can promote other stacking orders, such as the 2H configuration.^[^ ^60^ ^]^ Variants of CVD, including solution‐based approaches, are also explored to enhance the production of the 3R phase. Ambient pressure chemical vapor deposition (AP‐CVD) is a promising approach for synthesizing large‐scale horizontally and vertically aligned MoS_2_ on different substrates.^[^ ^61^, ^62^ ^]^ By controlling the growth zone configuration and process steps in CVD optimization, high‐quality MoS_2_ layers with different orientations can be achieved.^[^ ^63^ ^]^ CVD allows for the growth of high‐quality, large‐area 3R MoS_2_ films with precise thickness and uniformity that allows for precise control over the material's properties that leads to various optoelectronic applications.

The CVD synthesis of 3R‐MoS_2_ presents unique challenges, particularly due to the metastable nature of its ABC stacking configuration. These challenges are conceptually like those encountered in the growth of twisted transition metal dichalcogenides (TMDCs), such as twisted bilayer MoS_2_ or WS_2_, which require precise interlayer twist control and face high energy barriers for nonstandard stacking arrangements. Overcoming these barriers often requires advanced strategies, including space‐confined growth, modulated gas flow, or controlled precursor delivery.^[^ ^65^, ^66^, ^67^ ^]^ These approaches help disrupt standard stacking energetics and enable the growth of high‐quality, twist‐controlled TMDC films.

Similarly, the CVD growth of 3R‐MoS_2_ is challenged by the dominance of the 2H phase under typical growth conditions. The formation of the 3R phase requires strict control of several parameters: lower synthesis temperatures (typically 700–750 °C), optimized Mo:S precursor ratios, substrate influence or edge‐induced nucleation, and suppression of the thermodynamically stable 2H phase. These parameters help stabilize the ABC stacking required for the 3R polytype. However, as in twisted TMDCs, stacking faults, dislocations, and kinetic limitations often result in phase mixing or incomplete transformation. The yield and reproducibility of pure‐phase 3R‐MoS_2_ remain limited, mirroring the low success rate of twisted TMDC growth.

Thus, the synthesis of 3R‐MoS_2_ by CVD indeed shares fundamental difficulties with the CVD growth of twisted TMDCs, overcoming stacking energy barriers and precisely controlling nucleation kinetics to access thermodynamically unfavorable configurations. Further development of tailored CVD strategies, such as confined growth geometries or selective substrate interactions, may be required to reliably scale up 3R‐MoS_2_ synthesis for practical applications.

#### 3.2.3 Hydrothermal Synthesis

The first step involves mixing the necessary chemical ingredients, which typically include molybdenum sources (such as molybdenum oxide) and sulfur sources (such as thiourea or sulfur powder). These chemicals are dissolved in water to create a solution. The solution is then placed in a sealed autoclave. The autoclave is heated to a specific temperature, usually around 180 °C.^[^ ^68^ ^]^ This heating process is maintained for a certain period, often 24 h. The high temperature and pressure help the chemicals react and form the desired MoS_2_ structure. Similarly, in another report, the temperature and pressure conditions are optimized to favor the formation of the 3R phase over other phases such as 2H or 1T. The 3R phase is metastable and can be formed under specific conditions, which indicates that careful control of the synthesis parameters is necessary to stabilize the 3R phase. The synthesized 3R MoS_2_ typically exhibits a flower‐shaped morphology, which is a result of the hydrothermal conditions.^[^ ^69^ ^]^ The morphology is important as it can affect the electrochemical performance of the material when used in supercapacitors. Hydrothermal synthesis is relatively simple and can produce few‐layered MoS_2_ with good crystallinity. It also allows for the tuning of phase composition by adjusting precursor ratios.

#### 3.2.4 Solvothermal Methods

Solvothermal synthesis involves heating a precursor solution in a sealed vessel, promoting the formation of 3R‐MoS_2_ through controlled conditions such as temperature and pressure. This method can offer a balance between quality and scalability compared to CVD, although it may yield materials with more defects. Similar to hydrothermal synthesis, this method uses organic solvents instead of water. The precursors are dissolved in a solvent and heated in a sealed container to promote the formation of 3R MoS_2_. Typically, ammonium tetra thiomolybdate (NH_4_)_2_MoS_4_ or molybdenum trioxide (MoO_3_) “Mo” and thiourea (CH_4_N_2_S) as “S” source. Hydrazine (N_2_H_4_) as a reducing agent is often used to facilitate MoS_2_ formation. N, N‐dimethylformamide (DMF) is often chosen for its ability to dissolve precursors and promote selective growth of MoS_2_ on substrates. Alternative solvents that aid in controlling crystal growth are ethanol or ethylene glycol. The reaction occurs typically at 150–250 °C for a time interval of several hours (e.g., 12–24 h) in a sealed autoclave to allow for controlled nucleation and growth. Autogenous pressure develops due to solvent evaporation and reaction kinetics that allow MoS_2_ layers to nucleate and grow in the solvothermal medium. The phase purity (e.g., 3R MoS_2_ rather than the common 2H phase) is controlled by tuning reaction conditions. Substrates (e.g., graphene oxide, carbon supports) can be used to direct growth, preventing aggregation.^[^ ^70^ ^]^

Solvothermal synthesis can lead to different morphologies and phases of MoS_2_, including 3R, depending on the solvent and conditions used. The prominent challenges in this method are to avoid 2H‐phase contamination during rapid nucleation and maintain the metastability of 3R under prolonged heating (>24 h).

#### 3.2.5 Flux Growth

The use of carbonate or other flux materials can facilitate the growth of 3R‐MoS_2_ crystals. The salt flux approach was initially utilized by De Schulten,^[^ ^71^ ^]^ and is widely recognized as the most reliable approach for synthesizing bulk 3R‐MoS_2_ and has been adopted by various researchers.^[^ ^72^, ^73^ ^]^ Another method involves using a sulfur flux combined with high pressure, where sulfur exchange enables phase transformation. However, converting 2H‐MoS_2_ to 3R‐MoS_2_ requires extreme conditions (40−75 kbar, ≈2000 °C), making this process less practical than the carbonate flux method. Additionally, self‐propagating high‐temperature synthesis has been reported to produce impure 3R‐MoS_2_.^[^ ^74^, ^75^ ^]^ This method can help achieve larger crystal sizes; however, pure phase control remains a challenge. Rou et al. performed the synthesis using molybdenum oxide as the starting material.^[^ ^35^ ^]^ The synthesis was carried out in a carbonate flux, specifically using potassium carbonate. The reaction mixture got the addition of sulfur. The synthesis was conducted at varying temperatures: 550 °C, 650 °C, and 750 °C, among which 550 °C temperature was found to be the ideal synthesis condition for 3R‐MoS_2_, leading to a high concentration of the 3R phase and reduced crystallite sizes. This method is described as simple and scalable for producing 3R‐MoS_2_ materials.

#### 3.2.6 Chemical Vapor Transport (CVT)

CVT employs high temperatures to grow pure crystalline 3R‐phase MoS_2_ through recrystallization driven by transport agents within a temperature gradient. The method excels at producing single‐crystal samples that maintain controlled stacking sequences. Different dopant elements, including Re, Nb, Fe, and Co, allow control over CVT‐grown crystal phases when using Br_2_ as the transport medium. Through their research, Sigiro and Nasruddin showed that Re and Nb dopants promote the stability of 3R‐MoS_2_ crystal growth.^[^ ^76^ ^]^

A known ratio (9:20:1) of molybdenum (Mo), sulfur (S), and transporting agent molybdenum chloride (MoCl_5_), is prepared. The mixture is then put in an evacuated sealed quartz tube, allowing it to grow in an air and moisture‐free clean environment. A quartz tube is positioned within a three‐zone temperature furnace. Before crystal forming, the materials within the tube are preheated to a very high temperature (about 850 °C). In one segment of the tube, the temperature is elevated to 1080 °C (called the reaction zone), while another segment (the formation zone) is kept at a slightly lower temperature (920 °C). That temperature difference creates a gradient and allows the materials to flow and crystallize. The furnace is then cooled slowly to room temperature after 6 days at these temperatures. While the temperature drops, the precursors interact to produce 3R MoS_2_ crystals. Lastly, the resultant 3R MoS_2_ crystals are harvested from the growth area of the tube.^[^ ^30^, ^77^ ^]^ Mervin et al. found that 3R‐MoS_2_ crystals developed preferentially when they used Cl_2_ and I_2_ as transport agents.^[^ ^78^ ^]^ The CVT method produces crystals that serve as prime precursors for the mechanical exfoliation of MoS_2_ monolayers because of their convenient synthesis process and superior product quality.

#### 3.2.7 Interfacial Epitaxial Growth

Unlike conventional surface epitaxy, which struggles with phase purity and stacking control due to weak interlayer interactions and the small energy difference between the 2H and 3R phases, this interfacial epitaxy technique leverages a Ni‐transition metal alloy substrate to drive the upward precipitation of Mo and S atoms at a confined interface. This method, reported by Qin et al., allows for precise control over stacking order and thickness, enabling the uniform growth of 3R‐MoS_2_ films with thicknesses tunable from a few layers to over 15 000 layers.^[^ ^79^ ^]^

Wafer‐scale uniformity and crystallinity, high electronic quality, with room‐temperature carrier mobilities up to 190 cm^2^ V^−1^ s^−1^ in trilayers, enhanced nonlinear optical properties, including a five‐order‐of‐magnitude increase in difference frequency generation (DFG) efficiency under quasi‐phase‐matching conditions are some of the key advantages of this approach. This method overcomes many of the limitations discussed in our synthesis section and offers a scalable route to produce high‐purity 3R‐MoS_2_ single crystals, making it highly relevant for future integration into nanoelectronic and photonic devices.

### 3.3 Other Approaches

Combining methods mentioned above, such as integrating mechanical exfoliation with post‐synthesis treatments to optimize phase purity or morphology, can also be explored for producing 3R‐MoS_2_. Tailoring the synthesis route based on specific application requirements often leads to enhanced properties and performance. The salt‐templated synthesis method can produce both 2H and 3R polymorphs of MoS_2_. The stacking sequence difference leads to the 3R phase growth.^[^ ^80^ ^]^ Artificial monolayer manipulation involves folding, twisting, or restacking 2H‐MoS_2_ monolayers to produce 3R‐MoS_2_.^[^ ^81^ ^]^ In a less regulated manner of thermal treatment, Kumar et al. successfully facilitated the conversion of MoS_2_ monolayers into nanodomains of 3R‐MoS_2_. The researchers postulate that the phase transition transpires to mitigate the cavities produced due to the evaporation of sulfur atoms.^[^ ^82^ ^]^

## 4 Summary

These synthesis methods provide a range of options for producing 3R MoS_2_, each tailored to specific requirements such as purity, scalability, and cost‐effectiveness. They have their own set of benefits and challenges (**Table** **1**), allowing researchers to choose the most suitable approach for their specific applications. The top‐down methods are more suitable for exfoliating existing 3R MoS_2_ crystals, whereas bottom‐up methods enable controlled synthesis of 3R MoS_2_ films and nanostructures from precursor materials. Hydrothermal synthesis, CVD, CVT, and solid‐state reactions are all viable approaches, with each method contributing to the development of high‐quality 3R‐MoS_2_ for use in catalysis and optoelectronics. CVD stands out as the most favorable method for large‐scale production with excellent quality, while hydrothermal synthesis and solution‐based methods offer cost‐effective alternatives for smaller‐scale applications. Mechanical exfoliation remains a valuable tool for research purposes due to its ability to produce high‐purity materials. It's worth noting that the preparation of pure 3R phase MoS_2_ is challenging and often results in a mixture with 2H phase. The reliable synthesis of pure, high‐yield, and high‐purity 3R‐MoS_2_ is still one of the significant challenges in exploiting its full potential in applications. Continued research and optimization of these methods will further enhance the quality and scalability of 3R‐MoS_2_ production, enabling its usage in advanced optoelectronic, electronic, and catalytic applications.

**Table 1:** Showcasing different synthesis methods with their advantages and limitations.

Method	Advantages	Limitations	Ref.
CVD	High‐quality uniform films scalable for industrial applications	Expensive, complex reaction conditions, environmental concerns	[60]
Hydrothermal Synthesis	Simple, cost‐effective, controlled morphology	Limited scalability, long reaction times	[16]
Mechanical Exfoliation	High‐purity, defect‐free layers, no chemical contamination	Low yield, not scalable, inconsistent layer thickness	[44]
CVT	Produces high‐quality crystals	Requirement of longer time period and high temperatures.	[78]

## 5 Characterization

The investigation of 3R MoS_2_ has attracted considerable interest in the past few years because of its distinctive structural characteristics and possible applications across multiple domains, such as electronics, catalysis, and energy storage. Unlike its more commonly studied 2H counterpart, 3R MoS_2_ exhibits distinct characteristics that can be leveraged for innovative technological advancements. In this discussion, we will delve into the various properties of 3R MoS_2_, examining its basic structural, electronic, optical, and mechanical attributes, as well as their implications for practical applications.

### 5.1 Structural and Morphological Properties

**Figure 5:**
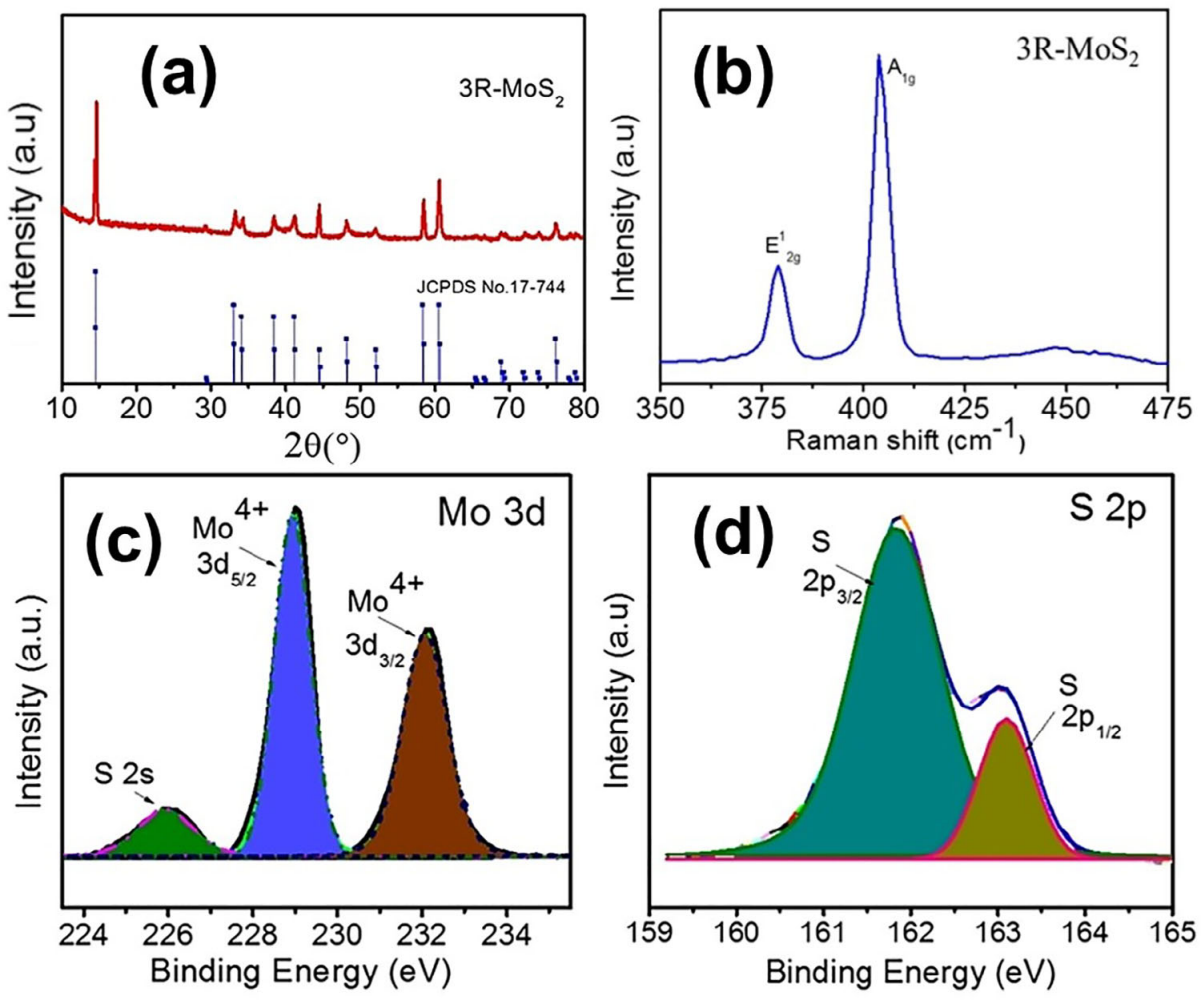
a) X‐ray diffraction (XRD) pattern, b) Raman spectra, highly resolved XPS spectra belonging to the c) Mo 3d, S 2s, and d) S 2p orbitals of 3R‐MoS_2_ flakes. Reproduced with permission.^[^ ^56^ ^]^ Copyright 2021, Wiley.

MoS_2_ occurs as numerous polytypes among the five MoS_2_ polymorphs—1T, 1T’, 1H, 2H, and 3R—the naturally occurring 2H and 3R phases, along with the metastable 1T phase, are particularly significant.^[^ ^83^ ^]^ The crystal structure of 3R‐MoS_2_ was determined more than six decades ago.^[^ ^84^ ^]^ The patterns obtained from powder X‐ray diffraction (XRD) of 3R‐ MoS_2_ flakes are portrayed in **Figure** **5**a, demonstrating the rhombohedral structure that aligns closely with JCPDS‐17‐0744. XRD patterns of 2H and 3R phases are largely overlapping due to their similar layered structures and interlayer spacings. However, minor differences in peak intensity and subtle shifts in 2*θ* values (e.g., for the (002) and (004) planes) can indicate the presence of 3R stacking. The broad and noisy nature of peaks in nanostructured or few‐layered MoS_2_ samples complicates this distinction. While 1T phase shows clear deviation in peak positions (e.g., lower 2*θ* for (002)), 3R and 2H phases require selected area electron diffraction (SAED) or high‐resolution transmission electron microscopy (HRTEM) for conclusive identification. 1T phase has characteristic J₁, J_2_, and J_3_ Raman modes (typically observed around 150–350 cm^−1^), which are absent in the 2H and 3R phases. However, 3R‐MoS_2_ and 2H‐MoS_2_ show very similar Raman features, particularly the in‐plane vibrational E^1^_2g_ (≈378 cm^−1^) and out‐of‐plane vibrational A_1g_ (≈403 cm^−1^) modes, as illustrated in Figure 5b making it difficult to distinguish them solely via Raman. Resonance shifts in 3R‐MoS_2_ inorganic fullerenes show a blue shift with increasing strain.^[^ ^85^ ^]^ Van Baren et al. used DFT calculations to study the Raman spectra, indicating that the shear mode peaks exhibit a redshift with a rise in the number of layers.^[^ ^30^ ^]^ Additionally, the peak separation (Δ) between E^1^_2g_ − A_1g_ mode frequency difference, which is slightly larger in 3R ≈25 cm^−1^ in bulk, decreases to 19 cm^−1^ for a single layer, compared to 2H enabling Raman‐based layer number measurement, but this is subtle and not conclusive on its own.^[^ ^10^, ^32^ ^]^ The chemical compositions of 3R‐MoS_2_ flakes are illustrated in Figure 5c,d. The highly resolved deconvoluted spectra (Figure 5c) of Mo 3d disclose two peaks at 229.0 and 232.1 eV, which correspond to the 3d_5/2_ and 3d_3/2_ binding energies of the Mo^4+^ state. Additionally, the peak at 225.9 eV is attributed to the 2s binding energy of S in the 3R‐MoS_2_ flakes. The peaks identified at 163.0 and 161.8 eV (Figure 5d) are associated with the S2p_1/2_ and S2p_3/2_ binding energies of sulfide ions (S^2−^).^[^ ^56^ ^]^ In summary, while Raman and XPS are excellent for identifying the 1T phase, the distinction between 3R and 2H requires more nuanced techniques, such as SAED or HRTEM, as their vibrational and chemical signatures are closely similar.

The noncentrosymmetric nature of 3R MoS_2_ enables applications in nonlinear optics and piezoelectric devices.^[^ ^86^ ^]^ The rhombohedral phase exhibits a larger interlayer distance, which can influence its electronic properties and interlayer coupling. The structural stability of 3R MoS_2_ under varying conditions is another significant aspect. Additionally, 3R phases are stabilized through van der Waals interactions between layers and can be theoretically modeled using density functional theory (DFT) that implements van der Waals corrections as necessary.^[^ ^87^ ^]^ Additionally, the ability to synthesize 3R MoS_2_ through various methods, including CVD and mechanical exfoliation, allows to produce high‐quality samples that retain the desired structural characteristics. The control over the synthesis process also enables the tuning of layer numbers, which can further modify the material's properties.

**Figure 6:**
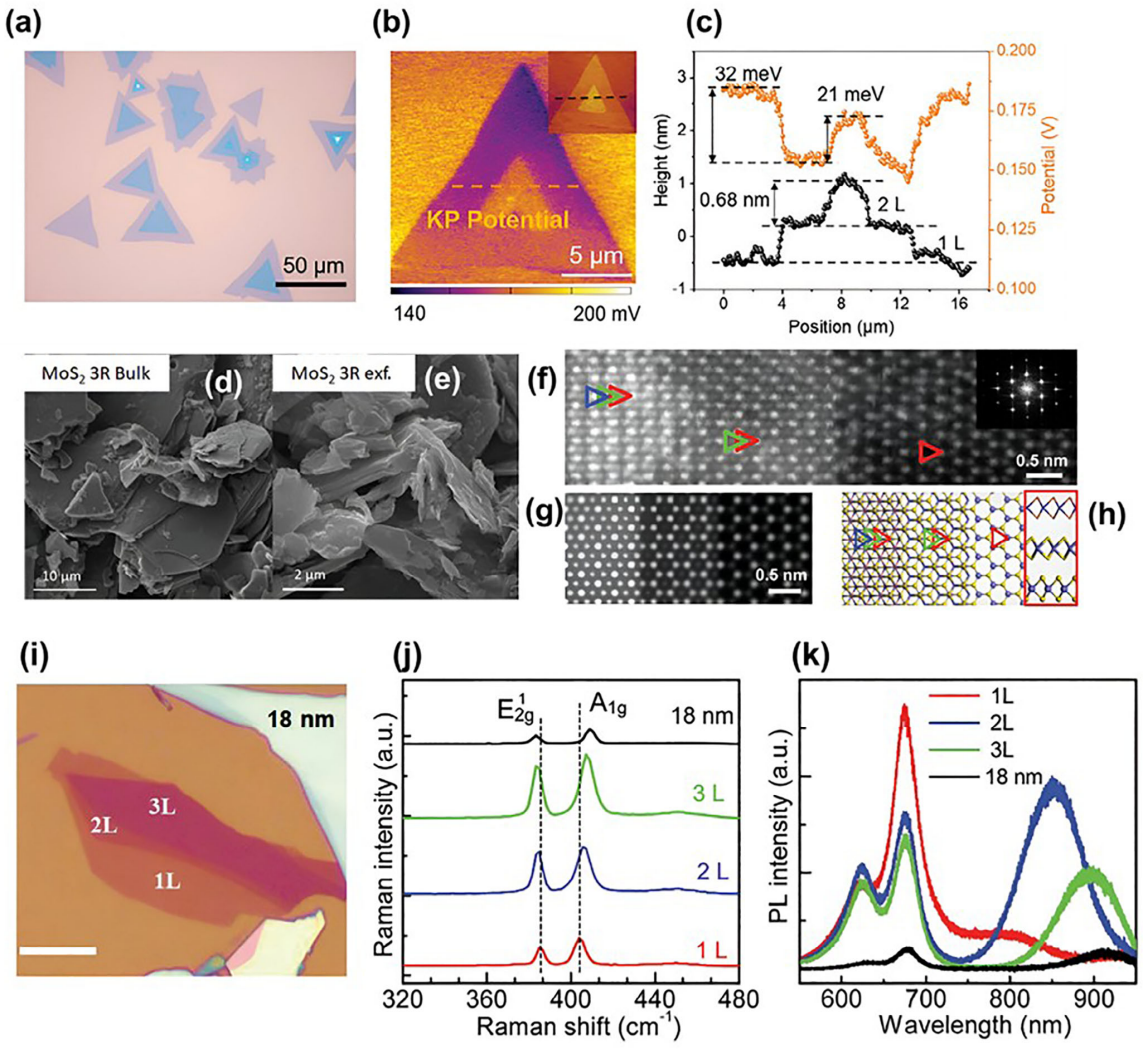
a) Optical microscopy image of a parallel‐stacked 3R‐MoS_2_ flake, b) scanning Kelvin probe microscopy (SKPM) image revealing the surface potential distribution across the 3R‐stacked MoS_2_, with the AFM topography shown in the inset, c) line profiles showing both surface potential (orange) and height (black) along the dashed line in (b). Reproduced with permission.^[^ ^60^ ^]^ Copyright 2024, Wiley. Scanning electron microscopy (SEM) images of 3R‐MoS_2_ in its (d) bulk and (e) exfoliated forms.^[^ ^88^ ^]^ f) Atomic‐resolution STEM‐ADF image displaying monolayer, bilayer, and trilayer 3R‐MoS_2_ from right to left, overlaid with corresponding diffraction patterns. Color‐coded triangles indicate the number of layers: red for monolayer, red and green for bilayer, and red, green, and blue for trilayer. g) Simulated STEM‐ADF image of 3R‐MoS_2_, h) Structural model corresponding to (g), clearly illustrating the atomic arrangement of various MoS_2_ phases. The red‐framed inset shows a side view of monolayer MoS_2_. i) Optical images showing 3R‐MoS_2_ flakes with varying thicknesses (1L, 2L, 3L, and 18 nm). j) Raman spectra comparing 3R‐MoS_2_ layers of different thicknesses. k) Photoluminescence (PL) spectra of 3R‐MoS_2_ with varying layer numbers, highlighting thickness‐dependent optical behavior. Reproduced with permission.^[^ ^77^ ^]^ Copyright 2024, Wiley.

**Figure** **6**a shows the optical microscopy image of a CVD‐grown bilayer 3R‐MoS_2_ flake exhibiting a parallel stacking configuration. The triangular morphology confirms high crystallinity and uniform stacking orientation, which is characteristic of the 3R phase synthesized under optimized reverse‐flow CVD conditions. Figure 6b presents the SKPM (scanning Kelvin probe microscopy) map of the same bilayer 3R‐MoS_2_, revealing a distinct surface potential distribution across the flake. The inset AFM topography verifies the bilayer structure with a step height of ≈0.68 nm between the layers. Figure 6c provides line profiles of both surface potential (orange) and height (black) along a dashed line in (b). The measured ≈21 mV surface potential difference between the stacked and bottom regions of the flake indicates the presence of intrinsic out‐of‐plane polarization, a hallmark of 3R stacking without inversion symmetry. This supports the existence of built‐in electric fields and aligns with the observed ferroelectric behavior in the bilayer 3R‐MoS_2_ system.^[^ ^60^ ^]^

Figure 6d shows the bulk 3R‐MoS_2_, which exhibits large, well‐defined layered sheets with lateral dimensions on the order of tens of micrometers. This morphology is typical of bulk MoS_2_ synthesized via high‐temperature, high‐pressure methods and reflects the high crystallinity and phase purity of the material. Figure 6e displays the exfoliated 3R‐MoS_2_, which reveals significantly downsized and thinner flakes with noticeable edge folding and surface wrinkling morphological features characteristic of successful exfoliation. The sheets now exhibit micron‐scale lateral dimensions and increased surface roughness, consistent with the exposure of catalytically active edge sites and defects beneficial for applications such as the HER. These images demonstrate the successful transition from bulk to few‐layered 3R‐MoS_2_ structures through chemical exfoliation, enhancing both the active surface area and potential functional performance.^[^ ^88^ ^]^

Figure 6f shows high‐resolution STEM‐ADF images of the monolayer, bilayer, and trilayer 3R‐MoS_2_ (from right to left), each overlaid with corresponding SAED diffraction patterns. The progressive inclusion of red, green, and blue triangles clearly marks the layer count and stacking order. These visualizations confirm the ABC stacking unique to the 3R phase, distinguishing it from the centrosymmetric 2H phase. Figure 6g provides a simulated (scanning transmission electron microscopy–annular dark field) STEM‐ADF image matching the experimental results in Figure 6f, reinforcing the interpretation of the 3R phase stacking and atomic arrangement. Figure 6h displays the structural model corresponding to Figure 6g, highlighting the positions of molybdenum (lavender spheres) and sulfur atoms (yellow spheres), along with a side‐view inset of monolayer MoS_2_. This model effectively visualizes the broken inversion symmetry that persists in multilayer 3R‐MoS_2_. The series of images in Figure 6f–h presents a comprehensive characterization of 3R‐MoS_2_, spanning its structural properties.^[^ ^77^ ^]^

### 5.2 Electronic Properties

**Figure 7:**
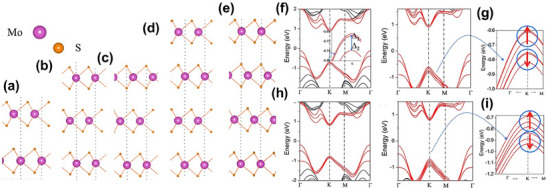
Multilayer 3R‐MoS_2_ crystal structures stacked in various configurations consist of a) 2‐layer‐Ab, b) 3‐layer‐ABA, c) 3‐layer‐ABC, d) 4‐layer‐ABAB, and e) 4‐layer‐ABCA‐stacking, band structures for MoS_2_ with 3‐layer ABA‐stacking f) without SOC and g) with SOC, similarly ABC‐stacking h) without and i) with SOC. Insets of Figure 6g and i show the band structure along K → Γ and K → M directions, beginning with the valence band top near the K point. The lengths for K → Γ and K → M segments represent 1/10th of the full path length in each direction.^[^ ^37^ ^]^

For 3R MoS_2_ the electronic structure differs significantly from the 2H phase. While 2H MoS_2_ is an indirect bandgap semiconductor, 3R MoS_2_ exhibits an indirect bandgap (1.416 eV) at the K point of the Brillouin zone.^[^ ^89^ ^]^ The bilayer 3R‐MoS_2_ exhibits an indirect bandgap, with the VBM located at the Γ point. DFT calculations, corroborated by optical spectroscopy studies, have confirmed this electronic structure feature. The CBM remains closer to the K point, indicating a Γ–K transition for the lowest energy excitons.^[^ ^90^, ^91^ ^]^ This property enhances its device applicability in optoelectronics such as light‐emitting diodes (LEDs) and photodetectors. Moreover, the absence of inversion symmetry in 3R MoS_2_ enables spin‐valley coupling, justifying its candidature for exciting spintronic applications.^[^ ^37^ ^]^ Another compelling aspect of 3R MoS_2_ is its electronic properties, particularly its band structure. The bandgap of 3R MoS_2_ has been exposed toward the layer number sensitivity, with bulk 3R MoS_2_ exhibiting a small indirect bandgap, while monolayer and few‐layer 3R MoS_2_ can display a transition to a direct bandgap.^[^ ^36^, ^92^, ^93^ ^]^ DFT studies have demonstrated that 3R phases exhibit enhanced electrical properties under certain conditions, such as strain or doping. Such modifications can adjust Fermi levels and optimize electronic device performance. The stacking order and SOC significantly influence the electronic properties, with evident separation of spin‐up and spin‐down channels.^[^ ^37^ ^]^ **Figure** **7**a–e illustrates multilayer MoS_2_ with various stacking orders used to simulate their impact on electronic properties. 3‐layer MoS_2_ sample's band structures for different stacking configurations, both with and without SOC, are shown in Figure 7f–i along the K → Γ and K → M directions. In 3R‐MoS_2_, the localization of wave functions at the K point is caused by the same‐direction layer stacking, which destroys inversion symmetry. Consequently, K point excitons in 2H‐MoS_2_ relax via intralayer valley relaxation between the K and K′ points rather than interlayer dispersion.

Moreover, the suppressed interlayer coupling at the K point is attributed to the contrast in the valley angular momentum between layers in 3R stacking. Due to the broken inversion symmetry and same‐direction (ABC‐type) stacking in 3R‐MoS_2_, the K‐valley wave functions become highly localized, minimizing interlayer hybridization and making interlayer hopping forbidden by symmetry.^[^ ^94^ ^]^ This unique combination of momentum‐space indirectness and layer‐decoupled valley physics gives rise to extended exciton lifetimes and pronounced valley coherence, making 3R‐MoS_2_ highly attractive for valleytronic and excitonic device applications. Because of its long exciton lifespan and restriction on interlayer hopping, 3R‐MoS_2_ automatically becomes an attractive choice for data storage applications. These theoretical predictions have been experimentally validated by Akashi et al.^[^ ^11^, ^95^, ^96^ ^]^ Furthermore, the electronic conductivity of 3R MoS_2_ is of particular interest. The unique stacking arrangement leads to a different density of states, which can enhance the material's conductivity. This property is vital for applications in field‐effect transistors (FETs), where high electron mobility is desired.

### 5.3 Optical Properties

Due to its indirect bandgap nature, 3R MoS_2_ demonstrates strong light absorption and enhanced PL compared to its 2H counterpart.^[^ ^97^ ^]^ It exhibits distinct excitonic peaks in PL spectra, making it valuable for optical sensors and photovoltaic (PV) applications. Figure 6i presents optical microscope images of 3R‐MoS_2_ with various thicknesses (1L, 2L, 3L, and ≈18 nm), showing distinct contrast variations that assist in layer identification. Figure 6j illustrates the Raman spectra of MoS_2_ with different thicknesses. Two prominent peaks, corresponding to the E^1^_2__g_ and A₁_g_ modes, shift with increasing layer number, indicating interlayer coupling effects and enabling precise thickness identification. Figure 6k shows PL spectra, where emission peaks near 620 and 680 nm are assigned to A and B excitons arising from direct transitions at the K point. Notably, the intensity and position of these peaks vary with layer number, reflecting the influence of interlayer interactions and quantum confinement.^[^ ^77^ ^]^ Together, these figures affirm the crystallographic phase, structural integrity, and distinct optical behavior of 3R‐MoS_2_, providing a strong foundation for its application in nonlinear optics and optoelectronic devices.

**Figure 8:**
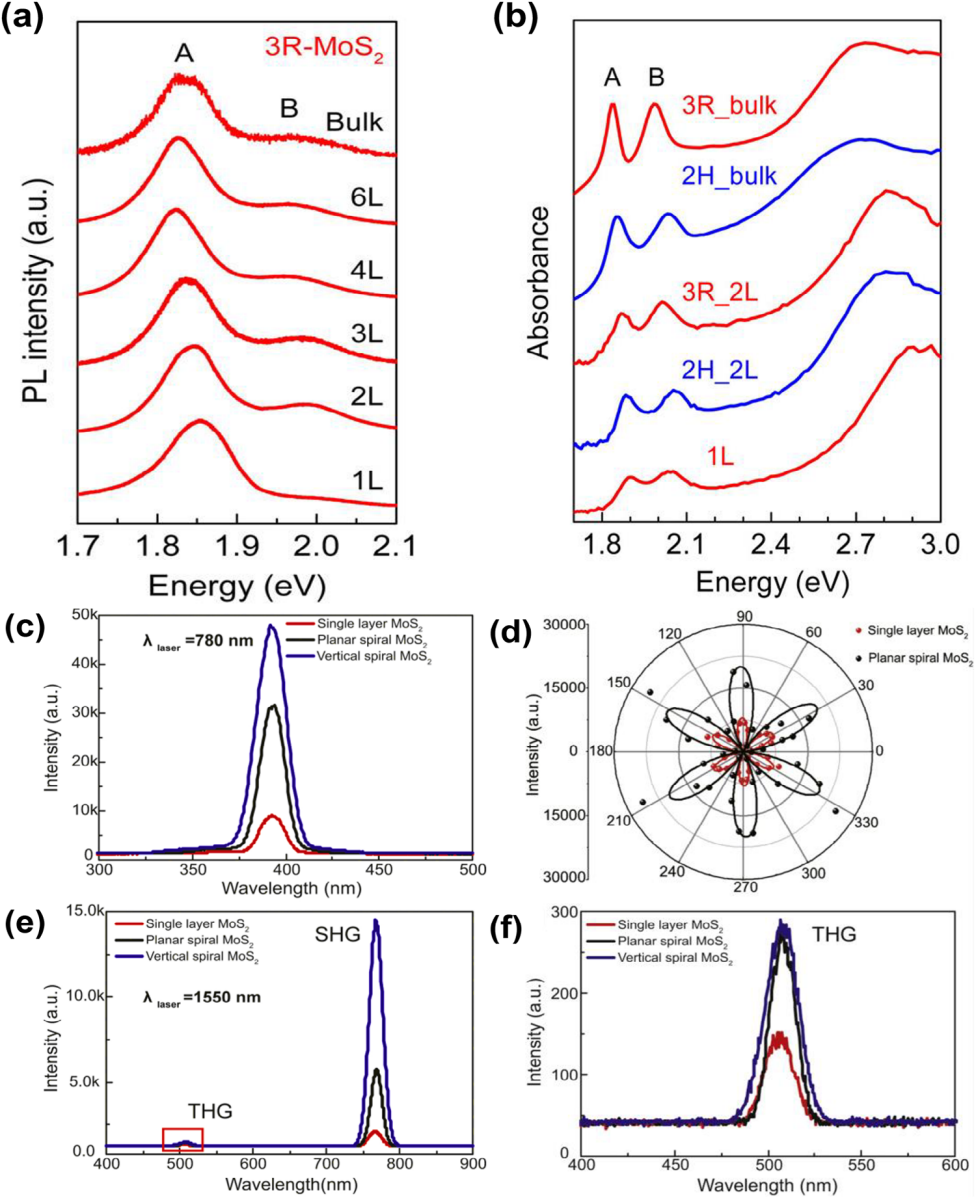
a) Normalized photoluminescence (PL) spectra of 3R‐MoS_2_ with varying layer numbers, b) absorbance spectra comparison between 2H‐ and 3R‐MoS_2_. Reprinted with permission.^[^ ^98^ ^]^ Copyright 2017 American Chemical Society. c) Second‐harmonic generation (SHG) measurements of single‐layer, planar, and vertical spiral MoS_2_ nanosheets using a 780 nm laser, d) angular dependence of the perpendicular SHG component for single‐layer (red dots) and spiral MoS_2_ (black dots), with theoretical fits (red and black lines), e) SHG and third‐harmonic generation (THG) of single‐layer, planar, and spiral MoS_2_ using a 1550 nm laser, and f) high‐magnification view of THG with the 1550 nm laser.^[^ ^32^ ^]^ © IOP Publishing. Reproduced with permission. All rights reserved.

The PL peak in 3R‐MoS_2_ remains largely unchanged with layer number, as depicted in **Figure** **8**a because it mainly originates from d‐orbitals of Mo shielded from energy exchanges among the layers.^[^ ^98^ ^]^ However, a slight blue shift and increased intensity occur with fewer layers due to quantum confinement. The fact that the PL intensity difference is constant throughout a range of layer numbers in circular dichroic PL investigations suggests that the valley information remains unchanged in layered 3R‐MoS_2_.^[^ ^10^ ^]^ Na et al. found the splitting of intralayer vibration (≈405 cm^−1^) occurred due to excitation of 1.96 eV, attributing this to interlayer interaction, known as Davydov splitting.^[^ ^99^ ^]^ In a few layers of 3R‐MoS2, two strong absorbance peaks (A and B) appear between 1.8 and 2.0 eV, which is attributed to d–d exciton transitions split by spin‐orbit coupling.^[^ ^10^, ^97^, ^100^, ^101^ ^]^ The deviation in energy between these excitons is about 150 meV, as determined by optical transmission spectroscopy and DFT calculations.^[^ ^95^, ^96^ ^]^ This energy difference remains unchanged with layer number or pressure (Figure 8b) due to decoupled interlayer wavefunctions.^[^ ^98^ ^]^ 3R‐MoS_2_, being noncentrosymmetric, exhibits strong second and third harmonic generation (SHG and THG). Figure 8c–f presents SHG behavior across various 3R‐MoS_2_ structures, including a polar plot of SHG intensities along with both SHG and THG for different layer numbers and stacking configurations and highly magnified THG plots.^[^ ^32^ ^]^ Redshift in SH signals by ≈1.5 meV for A and ≈10 meV for B exciton resonance frequencies per layer, respectively, is observed under the thin film limit (≈10 layers). SHG occurs at any number of levels, and because of reabsorption effects, the intensity of SH saturates at around 10 layers after expanding quadratically as the number of layers.^[^ ^32^, ^80^ ^]^

The 3R MoS_2_ demonstrate bulk nonlinear optical properties of |*d*_21_| =  500 ± 200 V^−1^, which further contributes to its potential in laser modulation and optical switching technologies.^[^ ^102^ ^]^ The complex dielectric functions (Figure 10a,b) show anisotropic behavior depending on the polarization of light for real and imaginary parts varying more significantly when aligned with the [010], [100], and [110] directions. A similar trend is observed in optical absorption (Figure 10c,d) up to 5.1 eV.^[^ ^81^ ^]^ Furthermore, the 2H phase's plasma frequency (5.4 eV) is greater than the 3R phase's (5.3 eV), suggesting that MoS_2_’s semiconductor characteristics can extend into metallic behavior.

### 5.4 Mechanical Properties

**Figure 9:**
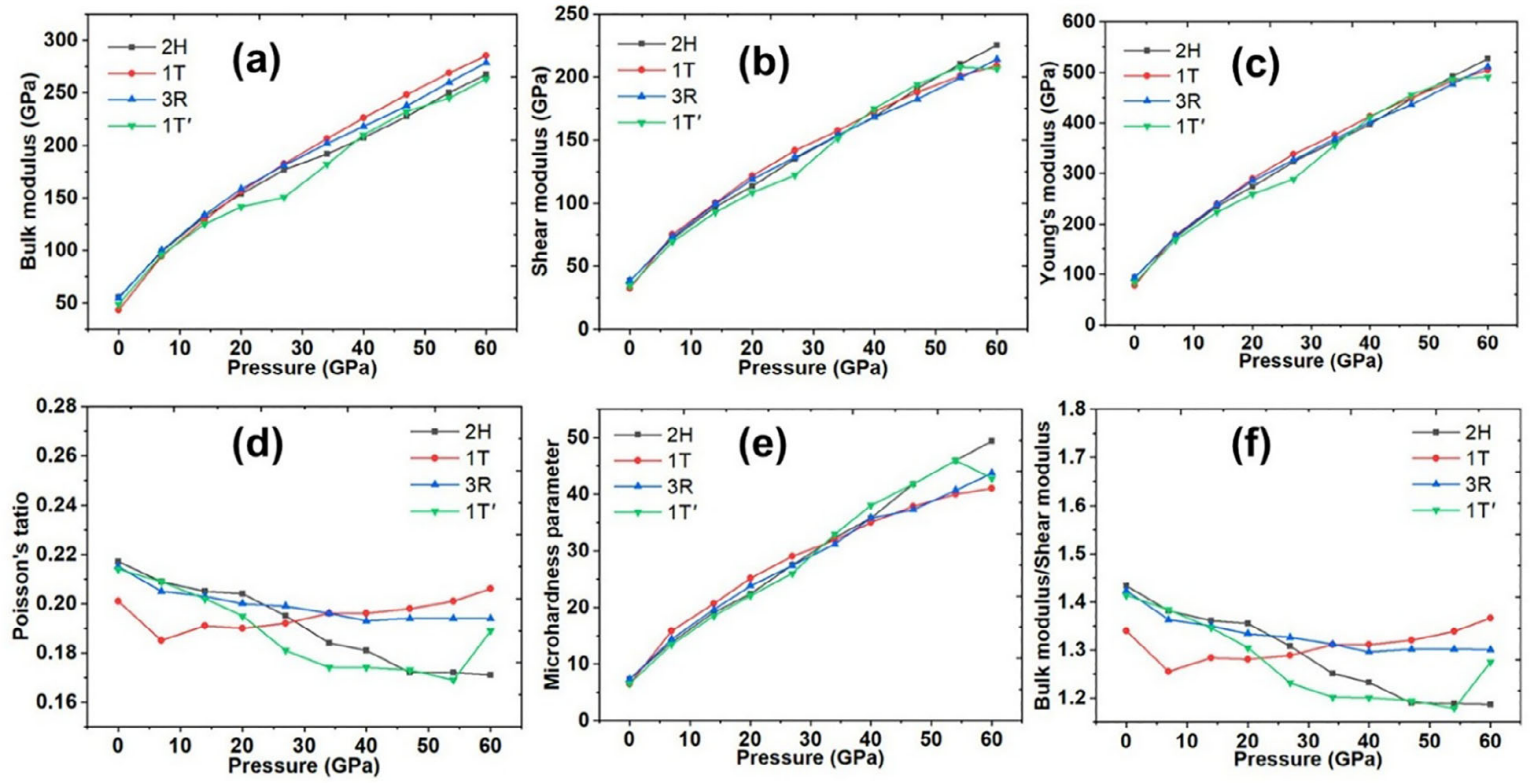
Elastic constants of various polymorphs (2H, 1T, 3R, and 1T′) of MoS_2_. a) Bulk modulus, b) shear modulus, c) Young's modulus, d) Poisson's ratio, e) microhardness, and f) bulk modulus/shear modulus ratio.^[^ ^34^ ^]^

Like other TMDs, 3R MoS_2_ exhibits high mechanical flexibility, making it suitable for flexible and wearable electronics. However, its interlayer interactions and stacking order affect its mechanical strength and shear modulus.^[^ ^103^ ^]^ The 3R phase is known for its slightly lower stiffness compared to the 2H phase, which can be advantageous in certain strain‐engineered applications.^[^ ^35^ ^]^ The mechanical properties of 3R‐MoS_2_ have been studied in comparison to other phases, as shown in **Figure** **9**. It exhibits a higher bulk modulus, Young's modulus, shear modulus, and microhardness compared to the 1T and 1 T phases, making it more stable for structural applications.^[^ ^34^ ^]^ The mechanical properties of 3R MoS_2_ are equally significant, particularly in the context of flexible electronics and composite materials. The layered structure of 3R MoS_2_ provides intrinsic flexibility, allowing it to withstand mechanical stress without compromising its structural integrity. This property is particularly advantageous for applications in flexible and wearable devices, where materials must endure bending and stretching. According to McClung, natural 3R‐MoS_2_ usually forms low aspect ratio particles that are elliptical or ball‐shaped. These particles are bendable, slow to float, have hydrophilic, oxide‐rich surfaces, and frequently contain significant concentrations of rhenium.^[^ ^104^ ^]^ Research has indicated that 3R MoS_2_ exhibits high tensile strength and resilience, making it suitable for use in composite materials that require durability and strength.

### 5.5 Thermal Properties

**Figure 10:**
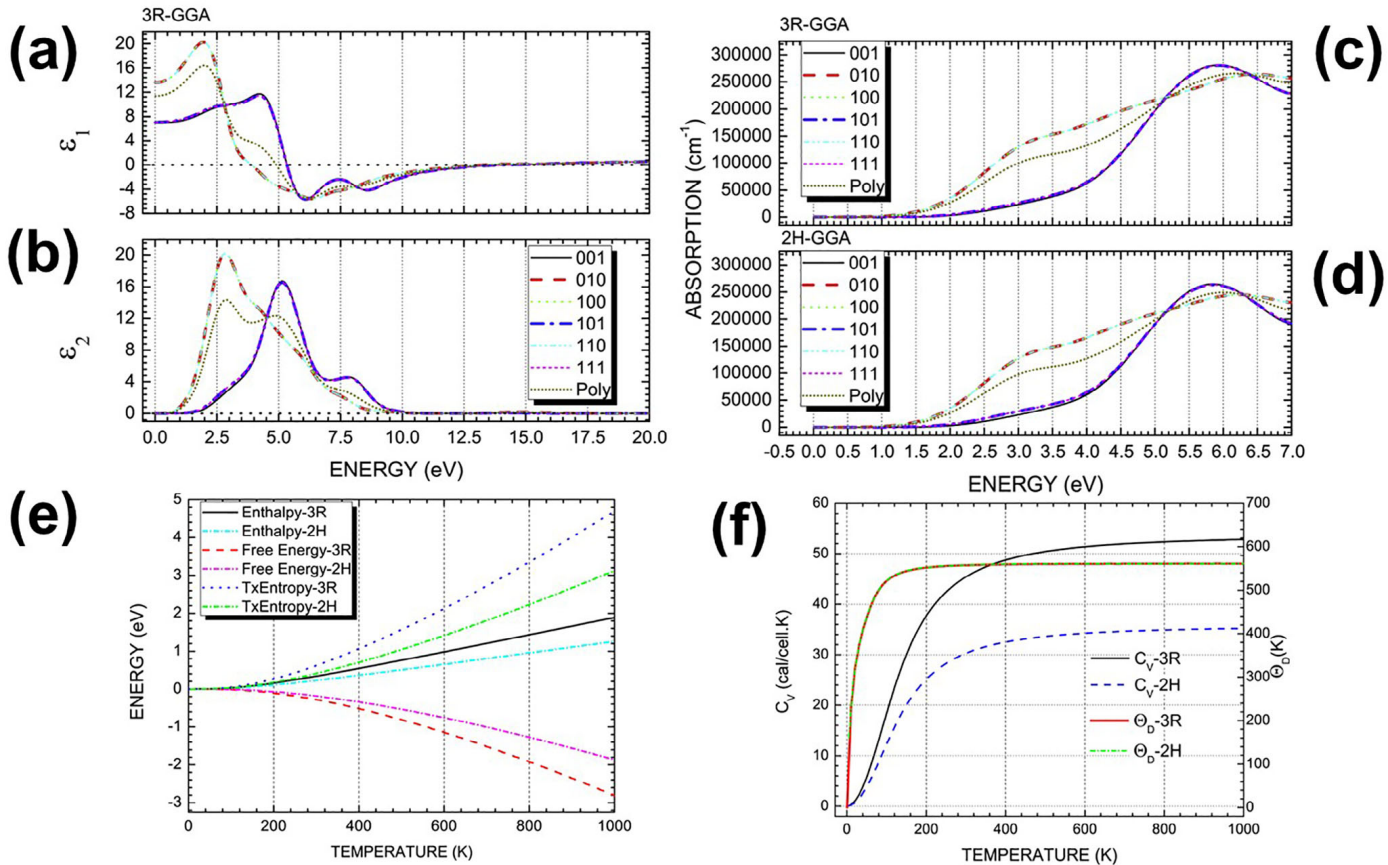
a) Real part (ε_₁_) and b) imaginary part (ε_2_) components of the GGA complex dielectric function for 3R‐MoS_2_. c,d) Optical absorption spectra of 3R‐MoS_2_ and 2H‐MoS_2_ close to the primary energy band gap, analyzed for different incidental light polarizations on different crystal planes including a polycrystalline sample (Poly), e) comparison of thermodynamic potentials for 3R‐MoS_2_ and 2H‐MoS_2_, including enthalpy (solid black for 3R‐MoS_2_, cyan for 2H‐MoS_2_), free energy (red for 3R‐MoS_2_, magenta for 2H‐MoS_2_), T: entropy (blue for 3R‐MoS_2_, green for 2H‐MoS_2_). f) Constant volume heat capacity (*C*_v_) of 3R‐MoS_2_ and 2H‐MoS_2_ as a function of temperature, alongside the Debye temperature (Θ_D_) variation with temperature (red solid and green short dash‐dot lines). Reprinted from publication^[^ ^86^ ^]^ Copyright 2017, with permission from Elsevier.

The thermal conductivity of 3R MoS_2_ is influenced by its stacking sequence and layer interactions.^[^ ^105^ ^]^ Due to its broken inversion symmetry, phonon transport in 3R MoS_2_ differs from the 2H phase, potentially leading to anisotropic thermal conduction.^[^ ^106^, ^107^ ^]^ This property is essential for thermal management in nanoelectronic devices, where efficient heat dissipation is crucial. The thermodynamic properties (entropy, enthalpy, heat capacity, free energy, and Debye temperature) in **Figure** **10**e,f suggest that as temperature increases, 2H‐MoS_2_ becomes slightly more energetically stable than 3R‐MoS_2_. However, for thermal insulation applications, the 3R‐MoS_2_ phase would be the preferred choice.^[^ ^86^ ^]^

### 5.6 Catalytic Properties

In addition to its electronic and mechanical attributes, 3R MoS_2_ has shown promise as a catalyst, particularly in HER and other electrochemical processes. The active sites within the 3R MoS_2_ structure can facilitate the adsorption and reaction of reactants, enhancing catalytic activity. The 3R phase of MoS_2_ demonstrates superior catalytic properties for HER compared to its 2H phase counterpart. Synthesized at 550 °C, 3R‐MoS_2_ shows a Tafel slope of 113 mV dec^−1^ and an overpotential of 0.52 V vs. RHE, significantly outperforming 2H‐MoS_2_ (overpotential reduction by 0.29 V).^[^ ^35^ ^]^ Pallellappa et al. reported that 3R‐MoS_2_ exhibits an overpotential of 670 mA cm^−^^2^, a Tafel slope of 172 mV dec^−1^, and a resistance of 2981 Ω. After 1000 cycles, the catalyst showed minimal change in electrocatalytic activity, demonstrating excellent stability. Additionally, it achieved a high (HER) rate of 675 µmol g^−1^ h^−1^, indicating strong photocatalytic performance.^[^ ^55^ ^]^ Furthermore, the catalytic activity of 3R‐MoS_2_ in hydrodesulfurization (HDS) has been shown to be superior to that of MoS_2_ derived from molybdenum naphthenate (MoNaph).^[^ ^108^ ^]^

While the 1T/2H phase has been found to exhibit higher photocatalytic activity due to the emergence of mid‐gap states, the 3R phase still shows significant potential in photocatalytic applications.^[^ ^109^ ^]^ Defects and doping strategies have been employed to further enhance the catalytic performance of 3R‐MoS_2_. For instance, heavy‐ion irradiation has been used to introduce defects in MoS_2_, leading to improved HER performance.^[^ ^110^ ^]^ Similarly, doping with transition metals such as Ni has been shown to enhance the catalytic activity of MoS_2_ in HDS reactions by creating more active sites.^[^ ^111^ ^]^ Theoretical studies have also highlighted the potential of doping elements such as B and C to modify the electronic structure of MoS_2_, thereby enhancing its catalytic activity.^[^ ^112^ ^]^ The potential for 3R MoS_2_ in energy‐associated applications, such as fuel cells and water splitting, is particularly compelling. As the world moves toward sustainable energy solutions, materials that can efficiently catalyze reactions for hydrogen production are in high demand. The unique properties of 3R MoS_2_ position it as a strong candidate for future research and development in this field.

## 6 Future Directions

Despite the promising properties of 3R MoS_2_, several challenges remain that must be addressed to fully realize its potential. One significant area of research is the scalability of synthesis methods. While techniques such as CVD show promise for producing high‐quality 3R MoS_2_, scaling up these processes for industrial applications remains a hurdle. Developing cost‐effective and efficient synthesis methods will be crucial for the commercialization of 3R MoS_2_‐based technologies. The incorporation of 3R MoS_2_ into polymer matrices can enhance the mechanical properties of the composite, leading to materials that are both lightweight and robust.

Additionally, further studies are needed to explore the long‐term stability and environmental impact of 3R MoS_2_ in practical applications. Understanding the degradation mechanisms and ensuring the material's stability under various operating conditions will be essential for its implementation in real‐world scenarios. Finally, interdisciplinary collaboration will be vital in advancing the research on 3R MoS_2_. Combining insights from materials science, chemistry, and engineering will facilitate the development of innovative applications and drive the integration of 3R MoS_2_ into existing technologies. Here's a concise comparison of the fundamental properties of 1T, 2H, and 3R polymorphs of MoS_2_, along with the metastable 1T' phase is mentioned in **Table** **2**:

**Table 2:** Evaluation of the fundamental properties of 1T, 2H, and 3R polymorphs of MoS_2_.

Property	1T (Metallic)	1T' (Distorted)	2H (Semiconducting)	3R (Semiconducting)
Crystal Structure	Trigonal (Octahedral	Distorted trigonal	Hexagonal (Trigonal prismatic)	Rhombohedral (ABC stacking)
Bandgap	Metallic (0 eV)	Semi‐metallic/small gap (≈0.05 eV)	Indirect: 1.2–1.8 eV (layer‐dependent)	Indirect: 0.8–1.81 eV (layer‐dependent)
Electrical Conductivity	High (metallic)	Moderate (semi‐metallic)	Semiconducting	Semiconducting
Stability	Metastable	More stable than 1T	Thermodynamically stable	Metastable (less common)
Layer Stacking	Single layer (Octahedral)	Distorted octahedral	ABA stacking	ABC stacking
Notable Properties	High catalytic activity Metallic conductivity	Topological insulator traits Quantum spin Hall effect	Strong photoluminescence High carrier mobility	Piezoelectricity Valley polarization
Ref.	[113, 114]	[115]	[116, 117]	[9]

## 7 Summary

In summary, 3R MoS_2_ presents a unique set of properties that distinguish it from its 2H counterpart, making it a material of great interest for various applications. Its structural stability, tunable electronic and optical properties, mechanical resilience, and catalytic potential underscore its versatility. As research continues to uncover the capabilities of 3R MoS_2_, it is poised to play a significant role in the future of electronics, catalysis, and energy storage applications. Addressing the challenges associated with synthesis, stability, and scalability will be critical in unlocking the full potential of this remarkable material. Through continued exploration and innovation, 3R MoS_2_ may well become a cornerstone of next‐generation technologies.

## 8 Applications of 3R MoS_2_ Phase

**Figure 11:**
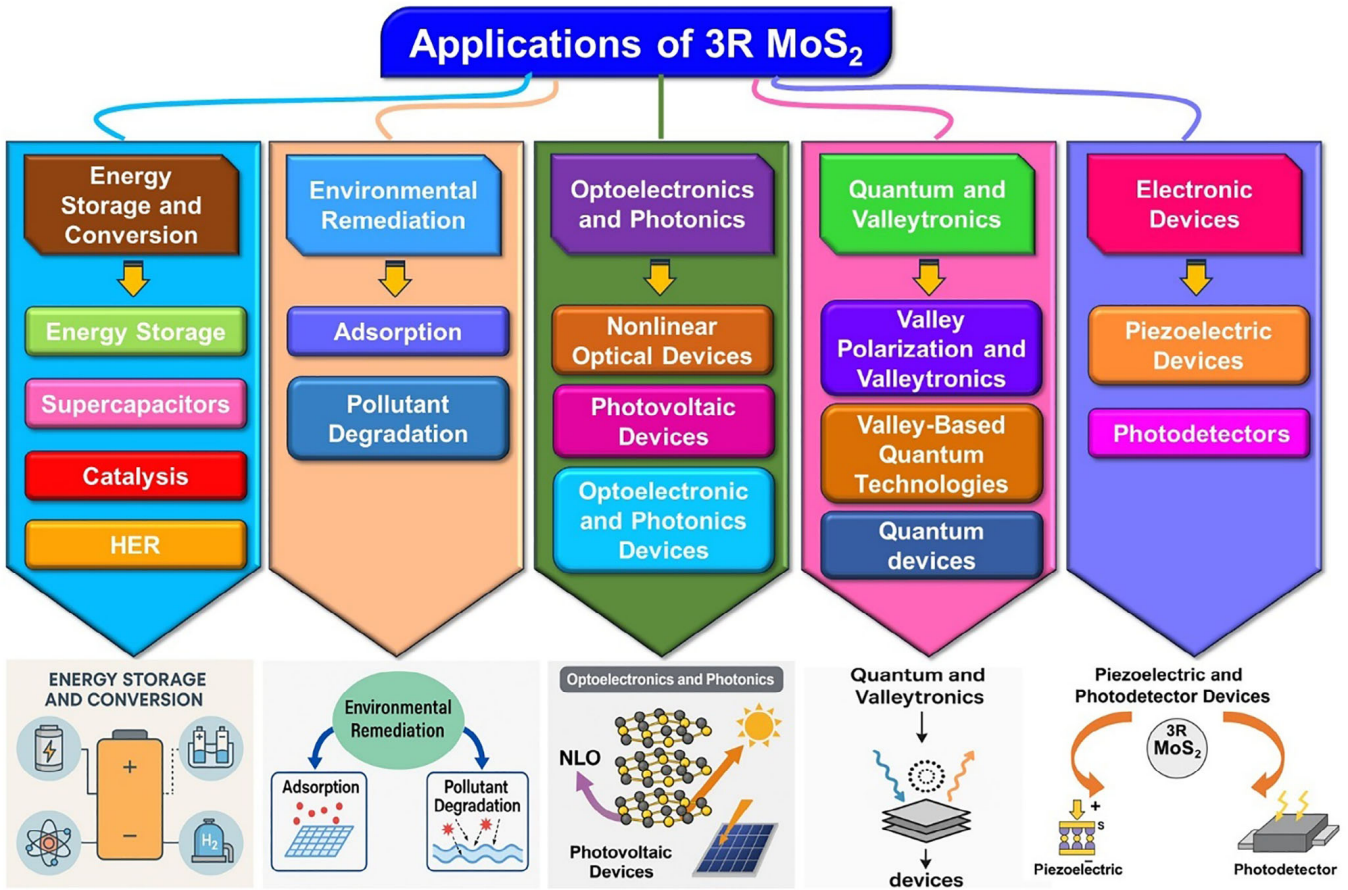
Schematic depiction of the applications of 3R‐MoS_2_ materials in different fields.

The 3R phase of MoS_2_ presents a unique rhombohedral crystal structure that differentiates it from the more prevalent 2H phase. This distinct noncentrosymmetric structure enhances its unique properties, such as indirect bandgap nature, strong nonlinearity, and PL, that provide opportunities for advanced device engineering. By virtue of these properties, this material expands its applications across various fields, particularly in advanced materials science, electronics, optoelectronics, energy storage, catalysis, and quantum technologies. Additionally, its mechanical flexibility and thermal properties make it suitable for flexible electronics and heat dissipation technologies. As research continues to explore, 3R MoS_2_ is expected to play a significant role in future technological advancements. In the following section, we have categorically mentioned the highlighted applications of 3R MoS_2_ in detail, supported by insights from recent research. These applications highlight the versatility of 3R MoS_2_ in various fields, including electronics, optoelectronics, environmental science, and energy storage. A schematic description of notable applications is given in **Figure** **11** below:

### 8.1 Energy Storage and Conversion

#### 8.1.1 Energy Storage

The layered structure of 3R MoS_2_ allows for efficient ion transport, making it an excellent candidate for supercapacitor electrodes. Its high surface area and electrical conductivity contribute to enhanced performance in energy storage devices. Research is ongoing to optimize its use in hybrid energy systems and supercapacitors. The 3R polymorph of MoS_2_ has been investigated as a potential cathode material for rechargeable aluminum‐ion batteries (AIBs). First‐principles calculations reveal that Al^3^⁺ ions can intercalate into the 3R‐MoS_2_ structure, offering a theoretical maximum specific capacity of 502.30 mAh g^−1^ and an average voltage range of 0.75–0.96 V. This makes 3R‐MoS_2_ a highly promising material for next‐generation energy storage systems.^[^ ^118^ ^]^

#### 8.1.2 Supercapacitors

The 3R phase of MoS_2_ has also shown potential in supercapacitor applications. When combined with other phases, such as 1T‐MoS_2_, it exhibits enhanced capacitance performance in various electrolytes. For instance, in a K_2_SO_4_ electrolyte, the 1T/3R phase achieves a specific capacitance of 590 F g^−1^ at a scan rate of 5 mV s^−1^. This highlights the suitability of 3R‐MoS_2_ for high‐performance symmetric supercapacitors.^[^ ^69^ ^]^ MoS_2_ demonstrates superior capacitive behavior, achieving a high specific capacitance of 513 F g^−1^ at 0.5 A g^−1^. It also exhibits the highest OER current density of 442.7 mA g^−1^ at a 10 mV s^−1^ scan rate. Additionally, it maintains long‐term stability, showing consistent performance over 1000 galvanostatic charge‐discharge cycles and during a 12‐h OER stability test.^[^ ^68^ ^]^ 3R MoS_2_ can be used as an electrode material in supercapacitors, benefiting from its high surface area and electrochemical properties, which contribute to improved energy density and cycling stability.

#### 8.1.3 Catalysis

**Figure 12:**
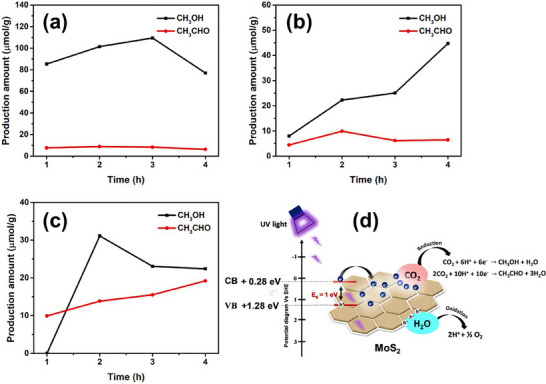
The CO_2_ reduction over 3R‐MoS_2_ sheets was evaluated in three different electrolytes: a) 0.5 m NaHCO_3_, b) 0.5 m NaOH, c) 0.5 m NaCl, and d) illustrates the photocatalytic CO_2_ reduction mechanism on MoS_2_ sheets, involving light absorption, electron‐hole pair generation, and surface catalytic reactions, leading to the formation of valuable chemical products. Reprinted from^[^ ^123^ ^]^ Copyright 2019, with permission from Elsevier.

3R MoS_2_ has shown superior performance in catalyzing HER, outperforming the 2H phase. It is particularly effective in converting into the metallic 1T phase, which enhances its catalytic activity. This makes it a promising candidate for applications in hydrogen production through water splitting and other electrochemical processes.^[^ ^119^, ^120^ ^]^ Additionally, it serves as an effective cocatalyst in petrochemical processes such as HDS.^[^ ^121^, ^122^ ^]^ The study by Geioushy et al.^[^ ^123^ ^]^ highlights the high photocatalytic performance of 3R MoS_2_ for CO_2_ photoreduction, producing liquid fuels and valuable chemicals such as methanol (CH_3_OH) and acetaldehyde (CH_3_CHO) as displayed in **Figure** **12**. These findings provide new insights into the potential of MoS_2_‐based catalysts for sustainable energy applications.

#### 8.1.4 Hydrogen Evolution Reaction (HER)

The 3R polymorph has demonstrated superior performance in the HER compared to the more common 2H phase. Studies show that 3R‐MoS_2_ can achieve a lower overpotential of −0.25 V vs. RHE, making it a highly efficient catalyst for hydrogen production.^[^ ^56^, ^88^ ^]^

### 8.2 Environmental Remediation

#### 8.2.1 Adsorption

**Figure 13:**
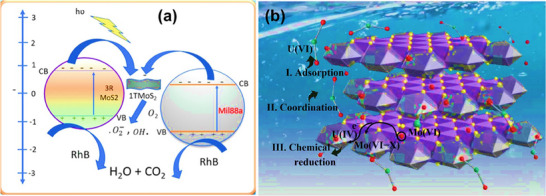
a) A schematic representation of the photocatalytic degradation mechanism of Rhodamine B (RhB) using the MMS‐30 photocatalyst under visible light, where photoexcited electrons and holes drive the breakdown of RhB molecules. Reprinted from,^[^ ^125^ ^]^ Copyright 2024, with permission from Elsevier. b) The proposed mechanism for U(VI) removal involves the PPy/3R‐MoS_2_ composite, where adsorption and redox interactions between U(VI) ions and the material's surface led to effective uranium uptake and reduction.^[^ ^124^ ^]^ Reproduced with permission from Springer Nature.

3R‐MoS_2_ has been explored for its adsorption and photocatalytic properties. When integrated with carbon cloth, it can effectively remove U(VI) from water with an adsorption capacity of 170–190 mg g^−1^ and a rapid equilibrium time of just 1 min.^[^ ^124^ ^]^ A schematic representation of the removal of U(VI) by PPy/3R‐MoS_2_ is depicted in **Figure** **13**b.

#### 8.2.2 Pollutant Degradation

The mixture of 3R‐MoS_2_ with further materials, for instance, exfoliated MIL88a, has been shown to enhance photocatalytic performance. This heterostructure exhibits high surface areas and efficient charge separation, making it effective for the degradation of pollutants in water.^[^ ^125^ ^]^ Its photocatalytic activity has been demonstrated in the degradation of organic dyes such as Rhodamine B (RhB) and Ciprofloxacin, achieving degradation efficiencies of up to 99.9%, as displayed in a scheme in Figure 13a. 3R MoS_2_ has shown significant piezo catalytic activity, particularly in the degradation of organic pollutants such as bisphenol A (BPA).^[^ ^126^, ^127^, ^128^, ^129^ ^]^ This application leverages its piezoelectric properties to enhance catalytic reactions under mechanical stress.

### 8.3 Optoelectronics and Photonics

#### 8.3.1 Nonlinear Optical Devices

**Figure 14:**
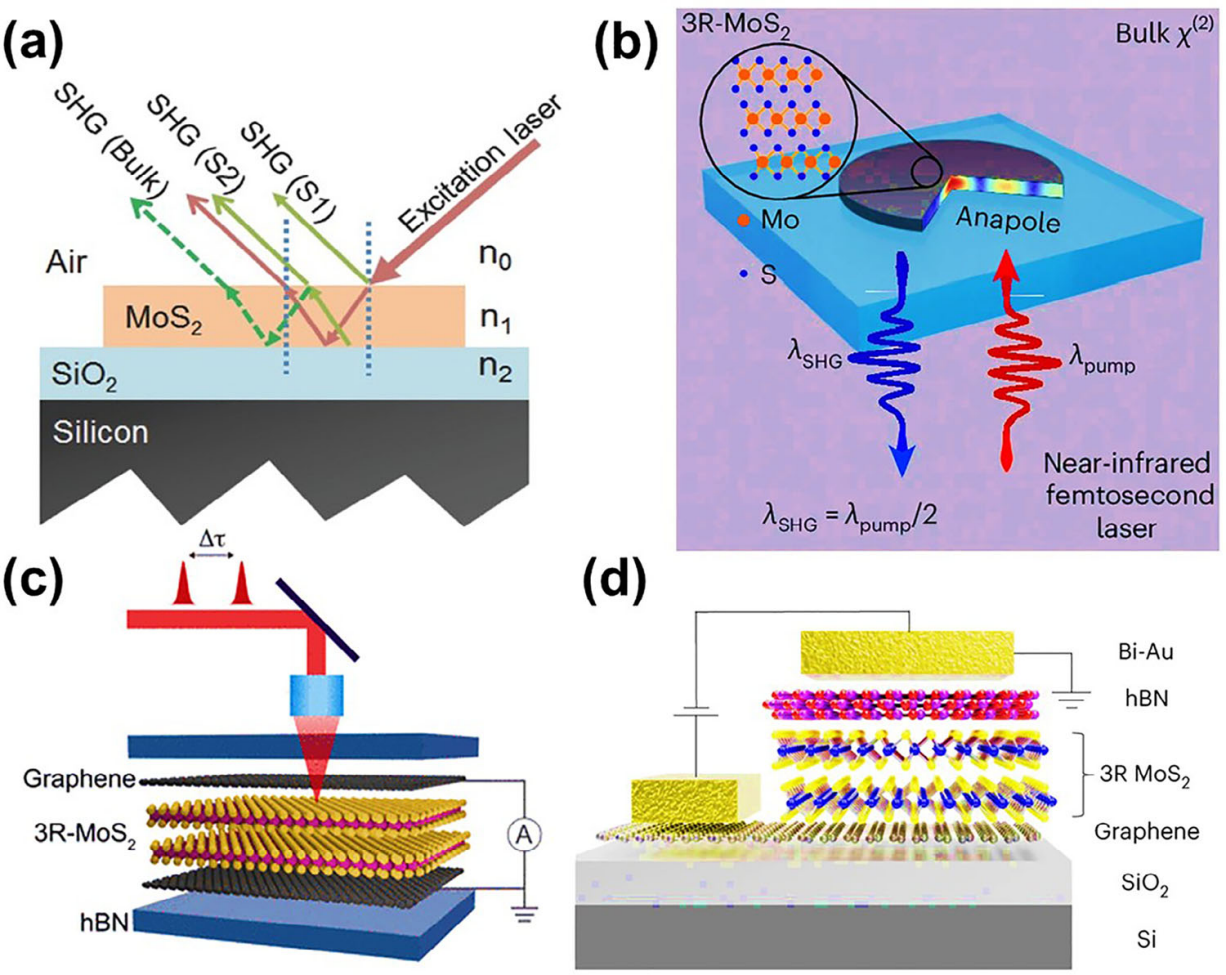
a) A diagram illustrates the geometry of the second harmonic generation (SHG) device. Reproduced with permission.^[^ ^77^ ^]^ © 2017 Wiley‐VCH Verlag GmbH & Co. KGaA, Weinheim. b) A schematic of the experiment demonstrates that a single 3R‐MoS_2_ nanodisk is excited by a near‐infrared femtosecond laser, effectively converting the input light into strong SHG output, highlighting its nonlinear optical efficiency.^[^ ^131^ ^]^ c) The schematic illustrates an ultrafast time‐resolved photocurrent measurement device. It features a bilayer or few‐layer 3R‐MoS_2_ flake sandwiched between graphene electrodes, and the entire structure is encapsulated in hexagonal boron nitride (hBN). The photocurrent is evaluated via graphene contacts using a lock‐in amplifier, while two collinear ultrafast laser pulses, separated by a time delay (Δ*τ*), are used to excite the device.^[^ ^132^ ^]^ d) The schematic shows a capacitive device structure composed of Bi/hBN/3R‐MoS_2_/graphene layers on a SiO_2_/Si substrate. A ≈10 nm thick hBN layer acts as an insulating barrier, effectively suppressing leakage currents and enhancing device performance.^[^ ^134^ ^]^ Reproduced with permission from Springer Nature.

The 3R polymorph of MoS_2_ is highly suitable for nonlinear optical applications due to its broken inversion symmetry and high refractive index.^[^ ^77^ ^]^ Bulk 3R‐MoS_2_ exhibits exceptional nonlinear SHG efficiency (**Figure** **14**a), nearly 100 times greater than that of bulk 2H‐MoS_2_. This overcomes the limitations of monolayer thickness and the weak nonlinear response of 2H‐MoS_2_, highlighting 3R‐MoS_2_’s potential as an ultrathin frequency‐doubling crystal and opening new avenues for integrated photonic applications. It has been used to fabricate ultrathin metasurfaces with atomically precise edges, demonstrating a significant enhancement in SHG over bulk materials, leading to quantum and classical nanophotonic applications.^[^ ^130^ ^]^ 3R MoS_2_ has been shown to have enhanced SHG efficiency (Figure 14b) compared to its 2H counterpart, making it a promising material for nonlinear optical applications, such as frequency doubling in laser systems.^[^ ^131^ ^]^

#### 8.3.2 Photovoltaic Devices

It has been demonstrated that the 3R‐MoS_2_‐based heterostructure device (Figure 14c) possesses a spontaneous PV effect with a picosecond‐fast photocurrent response and an external quantum efficiency of 10%. It is appropriate for high‐performance PV devices due to its rapid response, which equates to an inherent device bandwidth of about 100 GHz.^[^ ^132^ ^]^ Yang et al. also reported a spontaneous‐polarization‐induced PV effect in few‐layer 3R‐MoS_2_.^[^ ^133^ ^]^ This study demonstrated that rhombohedral stacking induces a strong depolarization field that drives intrinsic PV current at zero bias, with quantum efficiencies exceeding those of traditional ferroelectric PV devices. This work highlights the potential of 3R‐MoS_2_ for high‐performance, scalable, and voltage‐free photodetector applications.

#### 8.3.3 Optoelectronic and Photonics Devices

The unique optical properties of 3R MoS_2_ enable its use in photonic applications. Renji et al. studied a FET using a bilayer 3R‐stacked MoS_2_ flake, which demonstrated a high on/off current ratio of 10^8^ and a carrier mobility of approximately 8.6 cm^2^ V^−1^ s^−1^, indicating excellent electronic performance.^[^ ^21^ ^]^ Tilo et al. reported ferroelectric semiconductor transistors (FeS‐FETs) (Figure 14d) built on polarity switching in shear‐transformed (ST) 3R‐MoS_2_ epilayers, addressing challenges of mobile domain boundary formation. Their ultrathin bilayer ST‐3R‐MoS_2_ channel (1.3 nm) meets the requirements for sub‐3 nm CMOS nodes, offering low leakage, strong gate control, and simplified manufacturing. This work demonstrates the scalability and potential of 2D descending ferroelectric channels for next‐generation memory and logic devices.^[^ ^134^ ^]^ The first experimental demonstration of sliding ferroelectricity in bilayer 3R MoS_2_ was reported by Yang et al.^[^ ^65^ ^]^ This work revealed nonvolatile electrical polarization switching mediated by domain wall propagation in natural rhombohedral MoS_2_ bilayers. It established the mechanism of polarization switching via domain wall release and highlighted the potential of 3R MoS_2_ as a building block for programmable ferroelectric and optoelectronic devices. Yu et al. demonstrated that applying strain to 3R‐MoS_2_ leads to a nonlinear enhancement of the bulk photovoltaic effect (BPVE) by more than two orders of magnitude, highlighting the material's strong potential for next‐generation optoelectronic applications.^[^ ^135^ ^]^

### 8.4 Quantum and Valleytronics

#### 8.4.1 Valley Polarization and Valleytronics

In comparison to the centrosymmetric 2H phase, bilayer 3R‐MoS_2_ has demonstrated much greater valley polarization. This is explained by its indirect gap character, which essentially cancels out early valley polarization by allowing valley‐polarized excitons to relax into valley‐insensitive band edges. This property makes 3R‐MoS_2_ a robust platform for advancing valleytronics.^[^ ^25^ ^]^ Due to its lack of spatial inversion symmetry, odd‐layer 3R MoS_2_ is being explored for valleytronic applications, where information can be stored using different valleys in the material's band structure.^[^ ^25^ ^]^ Suzuki et al. demonstrated spin and valley polarization in 3R‐stacked MoS_2_ using SARPES, PL circular dichroism, and first‐principles calculations.^[^ ^10^ ^]^ Complementing this, Akashi et al. confirmed the absence of interlayer hopping for valley electrons through group‐theoretical analysis.^[^ ^96^ ^]^ Together, these findings highlight the ability to control valley excitations spatially in 3R‐MoS_2_, paving the way for advanced valleytronic device applications.

#### 8.4.2 Valley‐Based Quantum Technologies

MoS_2_ is a perfect material for valleytronics because its 3R phase maintains the valley degree of freedom irrespective of layer thickness. Research has shown that 3R‐MoS_2_ exhibits strong valley coherence, with a degree of linear polarization as high as 70% at 80 K. For the creation of upcoming quantum devices that use valley‐polarized photons, this characteristic is essential.^[^ ^62^, ^136^ ^]^

#### 8.4.3 Quantum Devices

The polar symmetry of 3R‐MoS_2_ enables independent control of layer and valley degrees of freedom through optical methods. This property has been theoretically proven using DFT and demonstrated experimentally through the observation of Wannier–Stark states.^[^ ^137^ ^]^ Such control is vital for developing next‐generation quantum devices.

### 8.5 Electronic Devices

#### 8.5.1 Piezoelectric Devices

**Figure 15:**
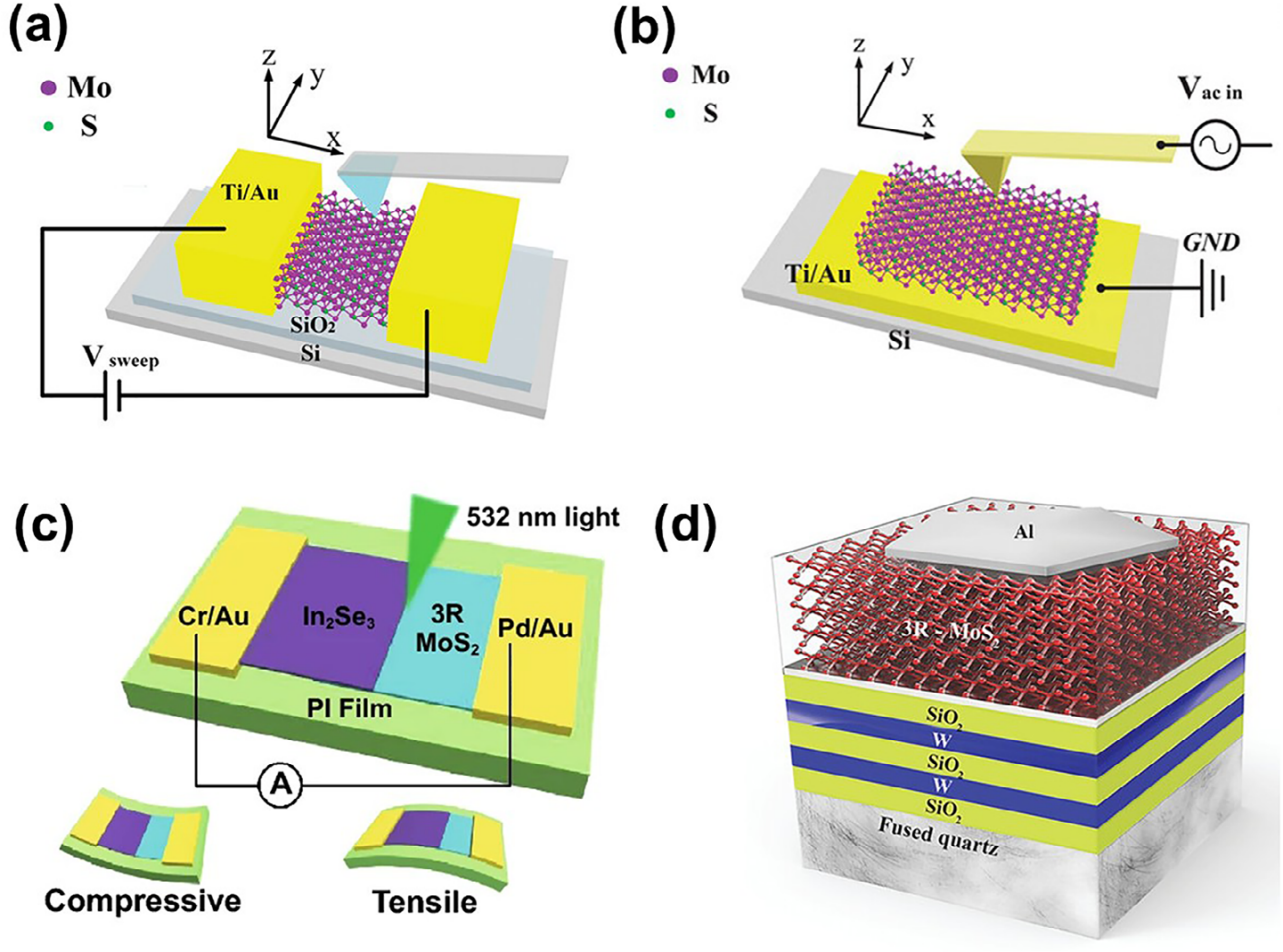
a) A schematic shows the PFM setup for measuring the out‐of‐plane piezoelectric coefficient (d_33_). b) Schematic representation of the PFM setup for in‐plane piezoelectric coefficient (d_13_)^[^ ^138^ ^]^ © 2022 Wiley‐VCH GmbH. c) A diagram depicts the 3R‐MoS_2_ and hexagonal α‐ In_2_Se_3_ heterojunction assembled on a flexible substrate.^[^ ^22^ ^]^ d) A 3D model illustrates the structure of a 3R‐MoS_2_‐based SMR^[^ ^140^ ^]^ © 2023 The Authors. Advanced Functional Materials published by Wiley‐VCH GmbH.

3R MoS_2_ demonstrates strong piezoelectric properties, making it ideal for piezoelectric devices. It can generate significant electrical signals when mechanically stressed, which is useful in sensors and energy harvesting applications. Hamida et al. discovered that 3R‐MoS_2_ flakes exhibit strong piezoelectric polarization when strained along the armchair direction. The flakes show significant amplitude and phase responses to applied electric fields. The piezoelectric coefficients were estimated to be approximately 0.9 pm V^cc^ (d_33_) for 18 nm thick flakes and 1.6 pm V^−1^ (d_13_) for 37 nm thick flakes.^[^ ^138^ ^]^ The piezoelectric parameter d_33_ represents the strain induced along the *z*‐axis (direction 3) when the electric field is also applied along the *z*‐axis, indicating out‐of‐plane polarization (**Figure** **15**a). In contrast, d_13_ refers to the strain induced in the *z*‐direction when the electric field is applied along the *x*‐axis (direction 1), showing cross‐axis piezoelectric behavior (Figure 15b). Among all single and multilayer forms, Dan et al. found that the 5‐layer 3R‐MoS_2_ structure had the greatest piezoelectric coefficient. This highlights the potential of multilayer 3R‐MoS_2_ for use in nano sensors and nanogenerators.^[^ ^139^ ^]^ Yang et al. successfully designed and fabricated novel 3R‐MoS_2_‐based solidly mounted resonators (SMRs) (Figure 15d) operating at frequencies above 20 GHz with compact dimensions (≈35 × 60 µm). The high operating frequency is attributed to the superior piezoelectric properties of submicron thick 3R‐MoS_2_. These findings suggest that 3R‐MoS_2_ is a strong candidate for next‐generation piezoelectric SMRs.^[^ ^140^ ^]^ Li et al. observed that monolayer MoS_2_ exfoliated from 3R‐stacked crystals exhibits out‐of‐plane switchable electric polarization under ambient conditions.^[^ ^141^ ^]^

#### 8.5.2 Photodetectors

Weifan et al. developed a self‐powered α‐In_2_Se_3_/3R‐MoS_2_ heterojunction photodetector (Figure 15c) with excellent visible and near‐infrared light response. Under a compressive strain of −0.26%, It obtained a detectivity of 6.2 × 10^10^ Jones and an ultrahigh photoresponsivity of 2.9 × 10^3^ A W^−1^.^[^ ^22^ ^]^ The study illustrates the application of mechanical strain to control carrier transport through the piezo‐phototronic effect at the heterojunction interface (**Table** **3**).

**Table 3:** Applications of 3R MoS_2_ across different fields.

Field	Application	Ref.
Energy storage	High‐capacity cathode material for aluminium‐ion batteries	[118]
Environmental remediation	Adsorption of U(VI) and photocatalytic degradation of organic pollutants	[124, 125]
Optoelectronics	Spontaneous photovoltaic (PV) effect and nonlinear optical devices	[21, 134, 135]
Quantum technologies	Valleytronics and robust valley polarization	[25, 136]
Material science	Enhanced mechanical properties and interlayer coupling modulation	[34, 37]

## 9 Summary

The 3R polymorph of MoS_2_ has demonstrated remarkable versatility across multiple fields, from energy storage and environmental remediation to optoelectronics and quantum technologies. Its unique properties, such as broken inversion symmetry, high surface area, and robust valley coherence, make it a promising material for addressing current technological challenges. As research continues to uncover its potential, 3R‐MoS_2_ is poised to play a pivotal role in advancing next‐generation devices and applications.

## 10 Conclusions and Future Perspectives

In summary, the 3R MoS_2_ polytype is a revolutionary substance that has the potential to enhance the functionality of gadgets of the future. Its unique structural properties, notably the alignment of layers and broken inversion symmetry, contribute to remarkable nonlinear optical characteristics that surpass those of conventional materials, facilitating compact device designs with high efficiency. Moreover, the versatility seen in its fabrication methods ensures that 3R MoS_2_ can be integrated into various photonic applications, thereby enhancing its practical viability. Recent explorations into its excitonic properties and phonon modes further substantiate its suitability for cutting‐edge technologies as research continues to uncover the full potential of 3R MoS_2_. It serves as both a foundational element that might completely alter the field of contemporary photonic devices and a basic substance for upcoming advancements. While significant advancements have been made in understanding the structural and optoelectronic properties of 3R‐MoS_2_, research on this polytype remains in its early stages compared to its 2H counterpart. The unique electronic structure, layer stacking, and noncentrosymmetric nature of 3R‐MoS_2_ present exciting opportunities, not only in optoelectronics but also in catalysis and energy storage. However, challenges such as scalable, high‐purity synthesis, and precise control over morphology and polytype must be addressed to unlock its full potential.

Because of its distinct electrical and optical characteristics, the 3R MoS_2_ polytype marks a substantial advancement in the creation of next‐generation device applications. As a two‐dimensional semiconductor, it exhibits exceptional tunability and flexibility, which are essential for advancing technologies in fields such as thermoelectrics and optoelectronics. For instance, recent studies have demonstrated promising potential in energy conversion applications, highlighting its relevance in the preparation and characterization of thermoelectric materials, including MoS_2_, using advanced methodologies. Furthermore, the emergence of twisted van der Waals structures and moiré superlattices opens new avenues for research, combining multiple disciplines and fostering innovative approaches to quantum phenomena in electronics.

**Figure 16:**
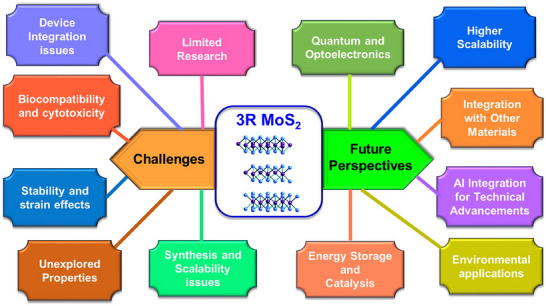
Schematic for displaying the various existing challenges and future perspectives of research in 3R‐MoS_2_ materials.

Future work should explore the catalytic and electrochemical behaviors of 3R‐MoS_2_ in greater depth, particularly for HER and intercalation‐based energy storage. The development of polytypic and polyatomic heterostructures may provide a pathway to optimizing these properties. Moreover, the influence of layer orientation on edge‐site activity and the resulting impact on reaction energetics remains a largely untapped field of study. The distinct band gap and optical features of 3R‐MoS_2,_ especially in few‐layer systems, also present promising avenues for photocatalytic applications that have yet to be fully explored. The unique morphologies and anisotropic conductivity of 3R‐MoS_2_ crystals suggest promising roles in electrochemical systems, especially where vertical charge transport and high edge‐site density are critical. With continued innovation in synthesis and integration strategies, 3R‐MoS_2_ stands poised to become a pivotal material in next‐generation catalytic and energy technologies. This integrative capability positions 3R MoS_2_ as a cornerstone for future explorations in materials science, enabling breakthroughs in device performance and addressing complex technological challenges, as shown in the schematic infographics in **Figure** **16**.

## Conflict of interest

The authors declare no conflict of interest.

## Data Availability

No primary research results, software, or code have been included, and no new data have been generated or analyzed as part of this review.
